# Dietary iron overload enhances Western diet induced hepatic inflammation and alters lipid metabolism in rats sharing similarity with human DIOS

**DOI:** 10.1038/s41598-022-25838-3

**Published:** 2022-12-10

**Authors:** Sakura Fujiwara, Takeshi Izawa, Mutsuki Mori, Machi Atarashi, Jyoji Yamate, Mitsuru Kuwamura

**Affiliations:** Laboratory of Veterinary Pathology, Osaka Metropolitan University, 1-58 Rinku-Orai-Kita, Izumisano, Osaka 598-8531 Japan

**Keywords:** Metabolic disorders, Obesity, Non-alcoholic fatty liver disease, Non-alcoholic steatohepatitis, Chronic inflammation

## Abstract

Hepatic iron overload is often concurrent with nonalcoholic fatty liver disease (NAFLD). Dysmetabolic iron overload syndrome (DIOS) is characterized by an increase in the liver and body iron stores and metabolic syndrome components. Increasing evidences suggest an overlap between NAFLD with iron overload and DIOS; however, the mechanism how iron is involved in their pathogenesis remains unclear. Here we investigated the role of iron in the pathology of a rat model of NAFLD with iron overload. Rats fed a Western (high-fat and high-fructose) diet for 26 weeks represented hepatic steatosis with an increased body weight and dyslipidemia. Addition of dietary iron overload to the Western diet feeding further increased serum triglyceride and cholesterol, and enhanced hepatic inflammation; the affected liver had intense iron deposition in the sinusoidal macrophages/Kupffer cells, associated with nuclear translocation of NFκB and upregulation of Th1/M1-related cytokines. The present model would be useful to investigate the mechanism underlying the development and progression of NAFLD as well as DIOS, and to elucidate an important role of iron as one of the "multiple hits” factors.

## Introduction

Non-alcoholic fatty liver disease (NAFLD) is the most common cause of chronic liver diseases worldwide today. It is strongly associated with metabolic syndrome (MetS) including obesity, type 2 diabetes mellitus (T2DM), dyslipidemia, and hypertension. The prevalence of NAFLD in the global population is estimated as 25% with a consistent rise in the past decade due to the increased number of MetS patients^1,2^. Most patients have a condition of hepatic triglyceride accumulation with minimal hepatocellular injury and inflammation (simple steatosis or nonalcoholic fatty liver [NAFL]), while some patients have hepatic triglyceride accumulation with more hepatocellular injury and inflammation (nonalcoholic steatohepatitis; NASH), which can progress to hepatic fibrosis/cirrhosis and finally to hepatocellular carcinoma (HCC). Epidemiological studies estimate that NASH prevalence is 59% and 6.7 to 30% among biopsied NAFLD patients and non-biopsied NAFLD patients, respectively, with 0.5% of them progressing to HCC^1^.

Although intensive efforts have found some therapeutic agents for NAFLD with an improvement of disease condition, unmet challenges still largely remain^3^. One of the major reasons for this difficulty is that the pathogenesis underlying the progression of NAFLD is quite complex and multifactorial. A “multiple-hits” hypothesis, stating that different etiological factors (e.g. insulin resistance, lipotoxicity, oxidative stress, alteration of immunity, and gut dysbiosis) are acting parallelly and synergistically during the development and progression of the disease, is widely accepted for understanding the pathogenesis of NAFLD^4,5^. Thus, establishment of preclinical animal models representing the full spectrum of human NAFLD is very challenging but is strongly required for development of effective therapeutics for NAFLD^6,7^.

Iron is an essential micronutrient for the body as it is necessary for vital biological processes such as oxygen transport, mitochondrial respiration, nucleic DNA synthesis, and cell signaling^8,9^. Excess iron accumulates in certain organs including the liver, heart, and endocrine glands, resulting in tissue injury via generation of reactive oxygen species by the Fenton reaction^9^. Iron dysregulation, manifesting as inappropriately low serum hepcidin levels and hyperferritinemia, is common in chronic liver diseases, whereas high level of serum and hepatic hepcidin has been reported in NAFLD since both obesity and diabetes are considered to increase hepcidin production^10,11^. Hepatic iron accumulation was present in 30–35% of NAFLD patients and several epidemiological studies have suggested an association between elevated body iron stores and MetS, NAFLD, and insulin resistance^12–14^. Furthermore, other studies have suggested that the presence and pattern of hepatic iron deposition are associated with advanced hepatic fibrosis, hepatocyte injury, and steatohepatitis in NAFLD^12,15,16^.

Dysmetabolic iron overload syndrome (DIOS) is a condition defined by an increase in the body iron stores associated with various components of MetS in the absence of any identifiable cause of iron overload. It is clinically diagnosed with hyperferritinemia, normal or moderately increased transferrin saturation, and the presence of MetS components^17–21^. DIOS and NAFLD are considered to be strongly correlated; approximately 50% of DIOS patients have NAFLD while more than 30% of NAFLD patients have DIOS^18,21^.

Although emerging evidences have suggested an association between NAFLD, iron overload, and MetS, the mechanisms how iron promotes progression of these metabolic diseases remain unclear^18^. Applicable animal model with iron overload, which represents hepatic and whole-body conditions of human NAFLD, would enable us to understand the influence of iron on metabolic diseases. We previously showed an increase in hepatic inflammation with upregulation of proinflammatory cytokines by dietary iron supplementation in a rat model of NAFLD^22^. However, the previous model had only a mild iron accumulation in the liver; it is insufficient to investigate the crosslink between NAFLD and DIOS. To our knowledge, there is no established model representing clinical and pathological phenotypes of both NAFLD with iron overload and DIOS. Therefore, this study is aimed to reveal the pathological role of iron overload in the pathogenesis of NAFLD, focusing on whether and how the iron overload affects the disease as one of the “multiple hits” factors, using a new animal model with long-term feeding of a Western diet with iron supplementation.

## Result

### Western diet feeding clearly induces obesity and hepatic steatosis irrespective of dietary iron content

**Figure 1:**
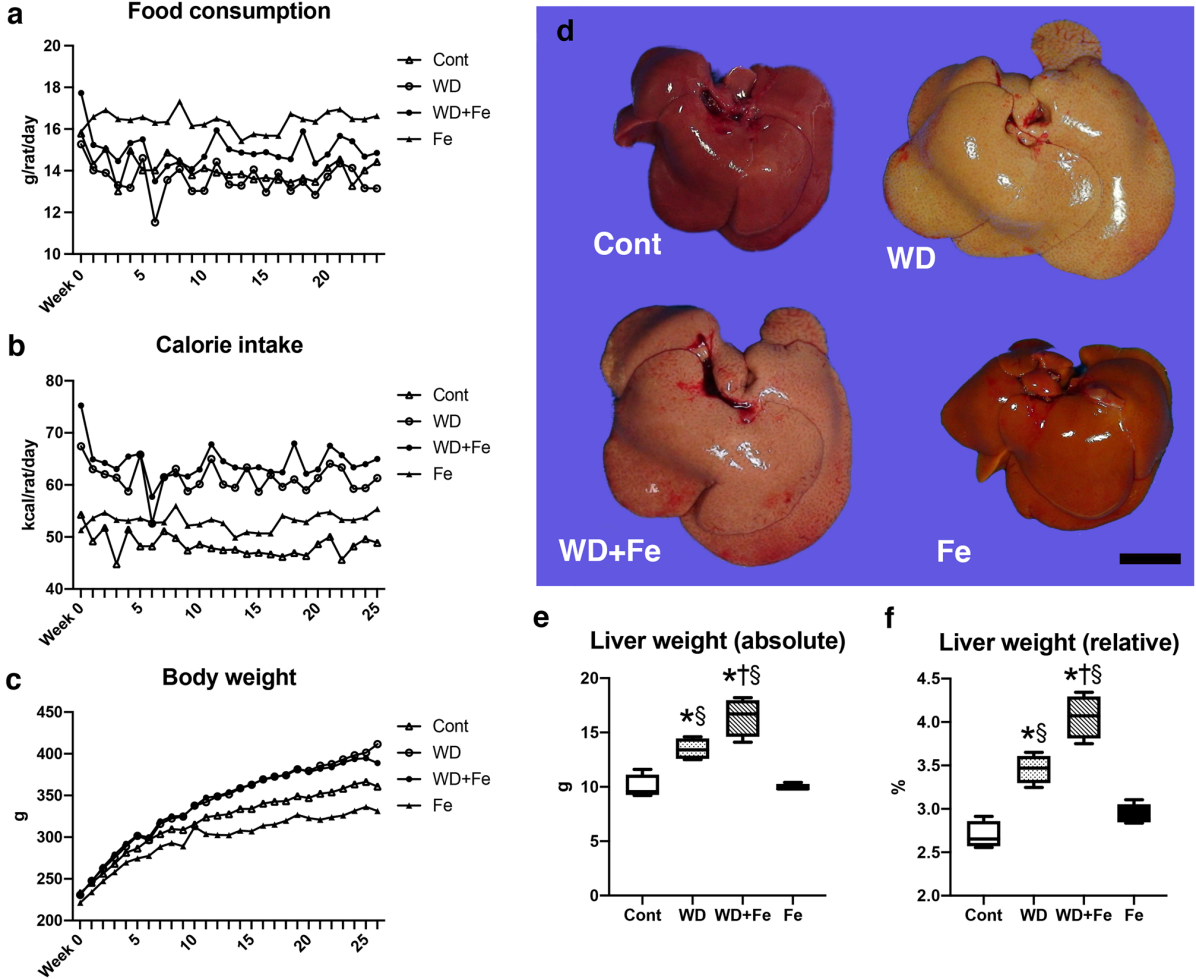
Temporal changes in (**a**) food consumption, (**b**) calorie intake and (**c**) body weight of control, WD, WD + Fe and Fe groups. Calorie intake was calculated by multiplying food consumption by estimated total calories of each diet shown in Supplementary Table 1. (**d**) Gross images of the livers of control, WD, WD + Fe, and Fe groups at week 26. Bar: 1 cm. (**e**) Absolute and (**f**) relative liver weight per 100 g body weight in control, WD, WD + Fe and Fe groups at week 26. Data are presented as box and whiskers (n = 4/group). *P < 0.05 versus Cont, ^†^*P* < 0.05 versus WD, and ^§^*P* < 0.05 versus Fe by one-way ANOVA followed by Tukey’s multiple comparison.

**Figure 2:**
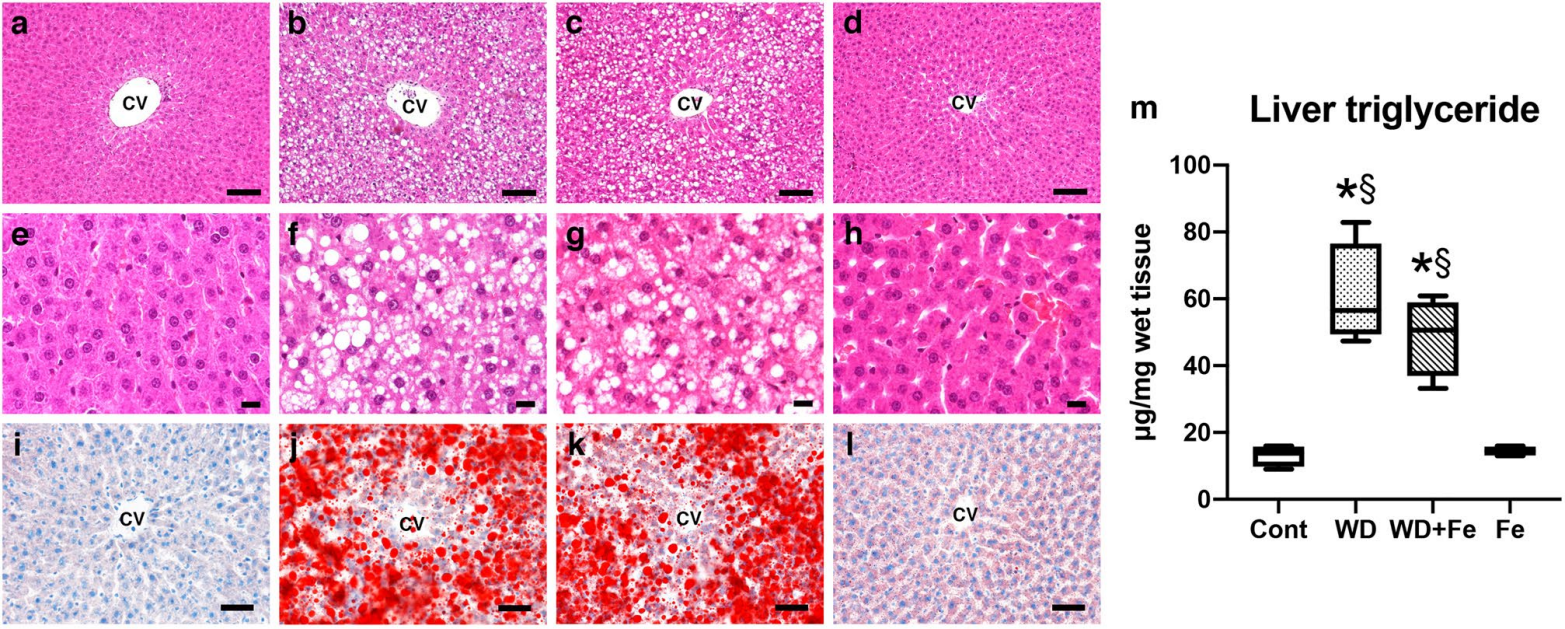
Histopathology of the liver in (**a**) control, (**b**) WD, (**c**) WD + Fe and (**d**) Fe groups at week 26. CV: central vein. Bar: 100 µm. (**e**–**h**) Higher magnification of **a**-**d**, respectively. Bar: 15 µm. Oil red O-stained sections in the liver of (**i**) control, (**j**) WD, (**k**) WD + Fe, and (**l**) Fe groups at week 26. Bar: 50 µm. (**m**) Hepatic triglyceride content at week 26. Data are presented as box and whiskers (n = 4/group). *P < 0.05 versus Cont, and ^§^*P* < 0.05 versus Fe by one-way ANOVA followed by Tukey’s multiple comparison.

Rats of Western diet (WD) and Western plus high-iron diet (WD + Fe) groups showed an increased body weight based on an increased calorie intake, compared with control (Cont) and high-iron diet (Fe) groups, respectively (Fig. 1b, c). Rats in high-iron diet (Fe) group had a lower body weight than Cont group, despite its higher food consumption and calorie intake (Fig. 1a–c). Increased appetite in rodents fed a high-iron diet is shown to be associated with cAMP-responsive element binding protein (CREB)-dependent downregulation of leptin in adipocytes^23^. The lower body weight in Fe group can be explained with a previous finding that iron overload has beneficial metabolic effects by upregulation of AMP-activated protein kinase activity in the skeletal muscle and liver ^24^. The livers of Cont and Fe groups presented a normal gross appearance. On the other hand, the livers of WD and WD + Fe groups had hepatomegaly with diffuse discoloration (Fig. 1d), suggestive of fatty liver. Both absolute and relative liver weights increased in WD and WD + Fe groups compared to Cont and Fe groups (absolute liver weight; *P* = 0.0055 for WD vs. Cont, *P* < 0.0001 for WD + Fe vs. Cont, *P* = 0.0049 for WD vs. Fe and *P* < 0.0001 for WD + Fe vs. Fe, relative liver weight; *P* = 0.0003 for WD vs. Cont, *P* < 0.0001 for WD + Fe vs. Cont, *P* = 0.006 for WD vs. Fe and *P* < 0.0001 for WD + Fe vs. Fe); they were higher in WD + Fe than in WD group (absolute liver weight; *P* = 0.0174, relative liver weight; *P* = 0.0025) (Fig. 1e, f). Histologically, the livers of Cont and Fe groups had no detectable abnormality (Fig. 2a, d, e, h). Diffuse microvesicular steatosis with scattered macrovesicular steatosis was observed in WD and WD + Fe groups, with its degree being similar between the two groups (Fig. 2b, c, f, g). The lipid vacuoles in hepatocytes of WD and WD + Fe groups stained red with oil red O (Fig. 2i–l). Consistent with the histological steatosis, hepatic triglyceride content increased in WD (*P* < 0.0001 vs. Cont, *P* = 0.0001 vs. Fe) and WD + Fe groups (*P* = 0.0012 vs. Cont, *P* = 0.0016 vs. Fe), with no significant difference between the two groups (Fig. 2m).

### Dietary iron supplementation induces hepatic iron overload with a marked iron accumulation in the sinusoidal macrophages/Kupffer cells

**Figure 3:**
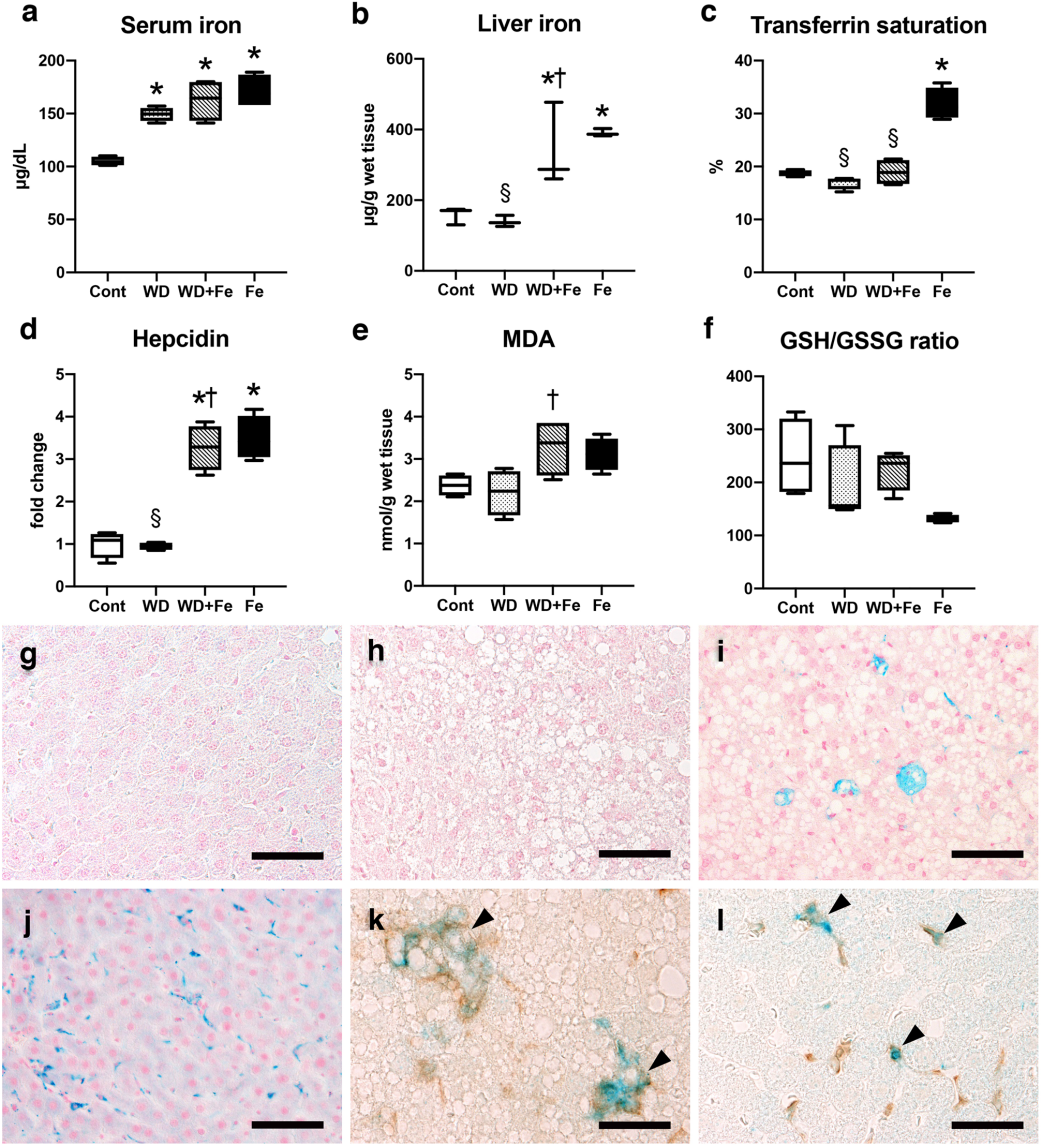
Blood biochemical data of (**a**) serum iron, (**b**) liver iron content, (**c**) transferrin saturation, and (**d**) hepatic expression of hepcidin mRNA at weeks 26. (**d**) Data were normalized to 18S rRNA and expressed as fold change from control group. (**e**) Hepatic content of malondialdehyde (MDA) at weeks 26, assayed by thiobarbituric acid‐reactive substances method. (**f**) Ratio of Hepatic content of glutathione (GSH)/oxidized glutathione (GSSG) at week 26. Data are presented as box and whiskers (n = 4/group). *P < 0.05 versus Cont, ^†^*P* < 0.05 versus WD, and ^§^*P* < 0.05 versus Fe by one-way ANOVA followed by Tukey’s multiple comparison. Images of Perls’ iron stain in the liver of (**g**) control, (**h**) WD, (**i**) WD + Fe and (**j**) Fe groups at week 26. Bar; 50 μm. IHC for Iba1 combined with Perls’ iron stain in (**k**) WD + Fe and (**l**) Fe groups at week 26. Arrowheads indicate iron accumulation (stained blue) in Iba1-positive macrophages/Kupffer cells (stained brown). Bar; 30 μm.

Serum and liver iron increased in Fe (serum iron; *P* < 0.0001, liver iron; *P* = 0.0071) and WD + Fe groups (serum iron; *P* = 0.0003, liver iron; *P* = 0.0262) compared with Cont group (Fig. 3a, b), associated with an upregulation of hepatic hepcidin (*P* < 0.0001 for Fe and WD + Fe vs. Cont) (Fig. 3d), the master regulator of systemic iron homeostasis^8,25^. Serum iron also increased in WD group compared with Cont group (*P* = 0.0025). Transferrin saturation, indicating binding capacity of the iron transporter transferrin to circulating free iron^25^, increased in Fe group compared with other 3 groups (*P* < 0.0001 vs. Cont, WD and WD + Fe) (Fig. 3c). Transferrin saturation did not change in WD and WD + Fe groups despite the increased serum iron; increased serum iron without increased transferrin saturation is a clinical feature of human DIOS^17,21^. Serum ferritin, a widely-used marker for body iron stores in humans^25^, did not significantly change between all groups; it was uncorrelated with hepatic iron stores (Supplemental Fig. S1a, b), as described elsewhere^26^. Perls’ iron stain revealed an iron accumulation in the sinusoidal cells and hepatocytes in WD + Fe and Fe groups compared with Cont and WD groups (Fig. 3g–j). The iron accumulation was more intense in the sinusoidal cells than in hepatocytes of both Fe and WD + Fe groups. Immunohistochemistry combined with iron stain demonstrated that iron was mainly accumulated in Iba1-positive macrophages/Kupffer cells in the sinusoid (Fig. 3k, l). Iron accumulation can induce oxidative stress and thus has often been implicated as one of the key factors for NAFLD progression^27,28^. Hepatic content of malondialdehyde (MDA), a commonly-used biomarker for oxidative stress (lipid peroxidation)^28^, increased in the WD + Fe group (*P* = 0.0403 vs. WD) (Fig. 3e), while hepatic glutathione (GSH)/oxidized glutathione (GSSG) ratio, an indicator of oxidative stress-antioxidant balance^29^, did not change significantly (Fig. 3f). These results suggested that oxidative stress, at least at the whole tissue level, does not play the major role in the pathogenesis of the fatty liver disease in this model.

### Western diet feeding induces dyslipidemia with partial alteration of hepatic insulin signal

**Figure 4:**
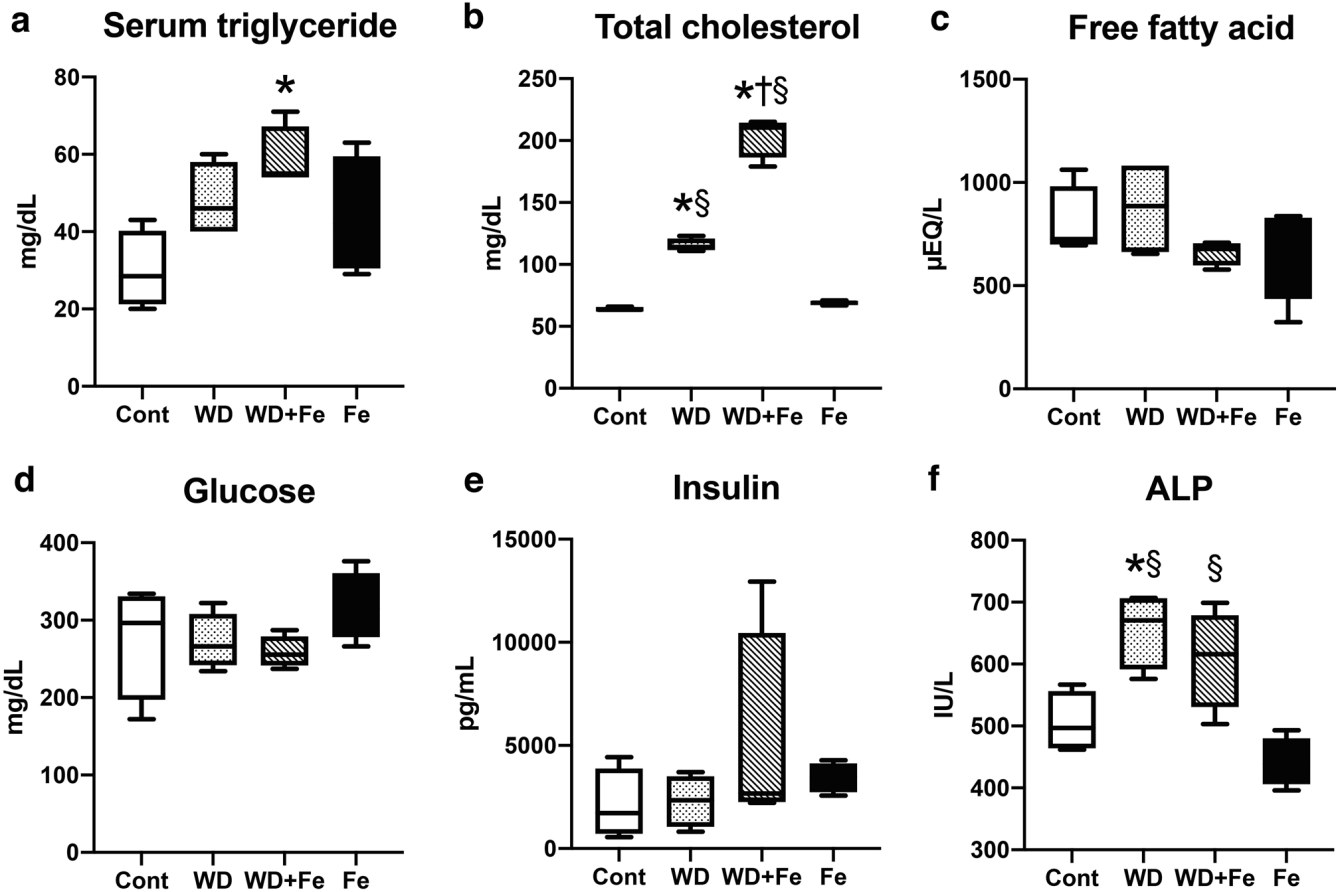
Blood biochemical data of (**a**) serum triglyceride, (**b**) total cholesterol, (**c**) free fatty acid, (**d**) glucose, (**e**) insulin, and (**f**) alkaline phosphatase (ALP) at weeks 26. Data are presented as box and whiskers (n = 4/group). *P < 0.05 versus Cont, ^†^*P* < 0.05 versus WD, and ^§^*P* < 0.05 versus Fe by one-way ANOVA followed by Tukey’s multiple comparison.

Serum triglyceride increased only in WD + Fe group (*P* = 0.0149 vs. Cont) (Fig. 4a). Serum total cholesterol increased in WD (*P* < 0.0001 vs. Cont and Fe) and WD + Fe (*P* < 0.0001 vs. Cont and Fe) groups compared with Cont and Fe groups; it was higher in WD + Fe than in WD group (*P* < 0.0001) (Fig. 4b). Serum free fatty acid, glucose, and insulin did not differ significantly between all groups (Fig. 4c–e); however, serum insulin value was very high in one of the four rats of WD + Fe group (12,949 pg/mL vs. 2107 ± 1698 pg/mL in Cont group). Serum alkaline phosphatase (ALP) increased by Western diet feeding (*P* = 0.0185 for WD vs. Cont, *P* = 0.0014 for WD vs. Fe, and *P* = 0.0093 for WD + Fe vs. Fe) (Fig. 4f). Serum aspartate aminotransferase and alanine aminotransferase did not increase in WD and WD + Fe groups and decreased in Fe group (Supplementary Fig. S2a, b).

**Figure 5:**
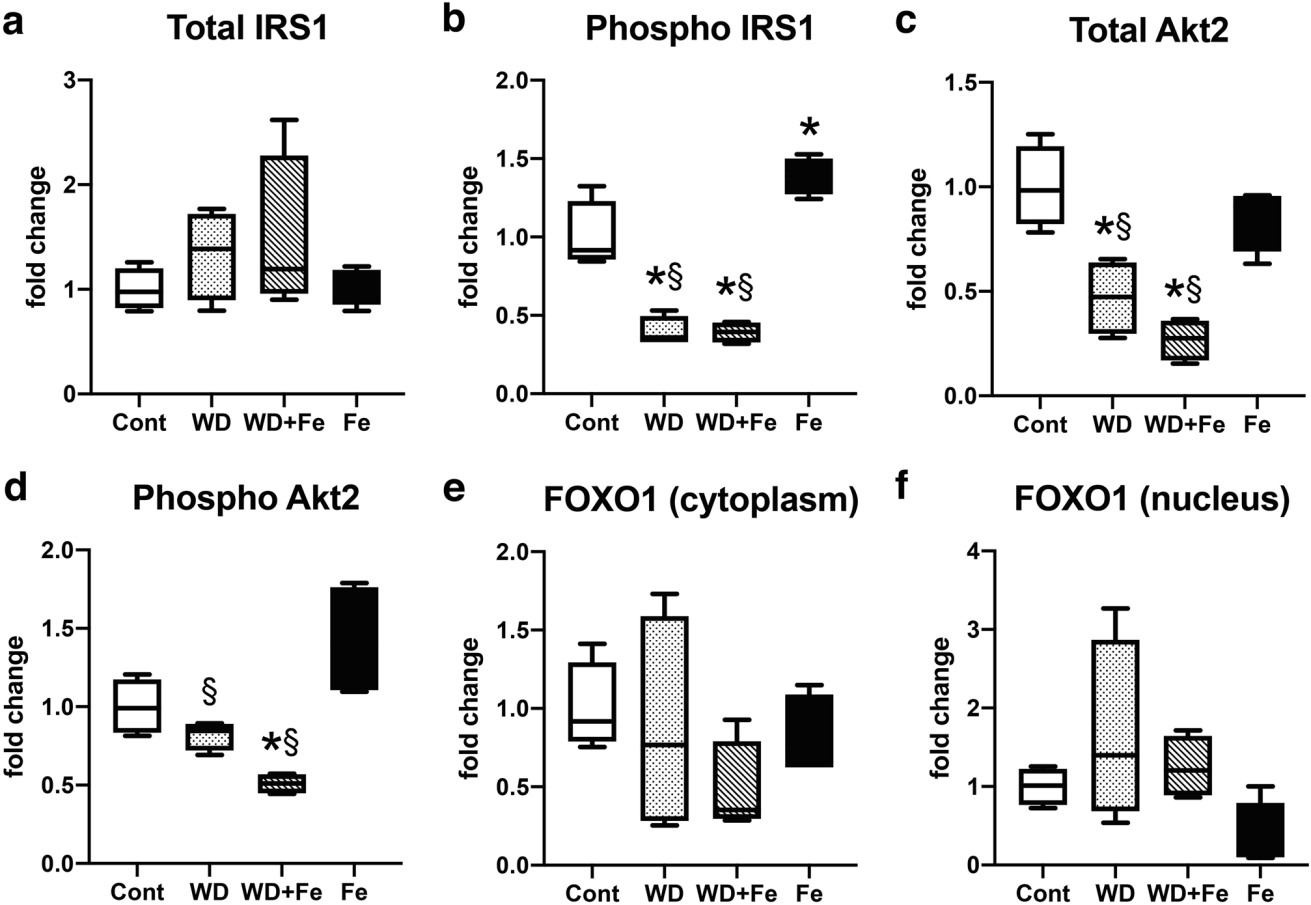
Hepatic expression of (**a**) total IRS1, (**b**) phospho-IRS1(Tyr 612), (**c**) total Akt2, (**d**) phospho-Akt2 from the whole liver tissue lysate at week 26. Hepatic expression of FOXO1 expression in the (**e**) cytoplasmic and (**f**) nuclear fractions at week 26. GAPDH and histone H2B were used for a loading control in the whole tissue/cytoplasmic and nuclear fraction, respectively. Data are expressed as fold change from control and are presented as box and whiskers (n = 4/group). *P < 0.05 versus Cont, and ^§^*P* < 0.05 versus Fe by one-way ANOVA followed by Tukey’s multiple comparison.

In the liver, insulin promotes phosphorylation of insulin receptor substrates (IRS1/2) at tyrosine residue. When IRS1 is phosphorylated at Tyr612, it mediates metabolic functions of insulin such as suppression of gluconeogenesis^30^. After the tyrosine phosphorylation of IRS1/2, Akt serine/threonine-protein kinases, especially Akt2, is activated by phosphorylation and then suppresses phosphorylation and nuclear translocation of the forkhead box protein O1 (FOXO1), a transcription factor regulating gluconeogenesis and glycogenolysis^30,31^. FOXO1 directly stimulates transcription of Pck1 and G6pc, the key hepatic gluconeogenic genes^30,31^. It is considered that MetS patients with insulin resistance may have inhibition of tyrosine phosphorylation of IRS1, leading to downregulation of Akt2 phosphorylation; the suppression of Akt2 activation keeps FOXO1 in the nucleus, resulting in persistent or excessive activation of hepatic gluconeogenesis^32^. In this study, hepatic expression of total IRS1 did not differ significantly between all groups (Fig. 5a) while tyrosine phosphorylation in IRS1 (Tyr612) decreased in WD (*P* = 0.0002 vs. Cont and *P* < 0.0001 vs. Fe) and WD + Fe (*P* = 0.0002 vs. Cont and *P* = 0.0087 vs. Fe) groups compared with Cont and Fe groups (Fig. 5b). Expression of total and phosphorylated Akt2 also decreased in WD (total Akt2; *P* = 0.0027 vs. Cont and *P* = 0.0259 vs. Fe, phosphorylated Akt2; *P* = 0.007 vs. Fe) and WD + Fe (total Akt2; *P* = 0.0002 vs. Cont and *P* = 0.0013 vs. Fe, phosphorylated Akt2; *P* = 0.0272 vs. Cont and *P* = 0.0002 vs. Fe) (Fig. 5c, d). Nuclear translocation of FOXO1 did not change significantly between the four groups (Fig. 5e, f). Additionally, hepatic expression of Pck1 and G6pc genes was downregulated in WD and WD + Fe groups (Supplementary Fig. S3a, b). These data suggest that insulin signal pathway is at least partly altered in the liver of WD and WD + Fe groups.

### Dietary iron overload enhances hepatic inflammation with nuclear translocation of NFκB

**Figure 6:**
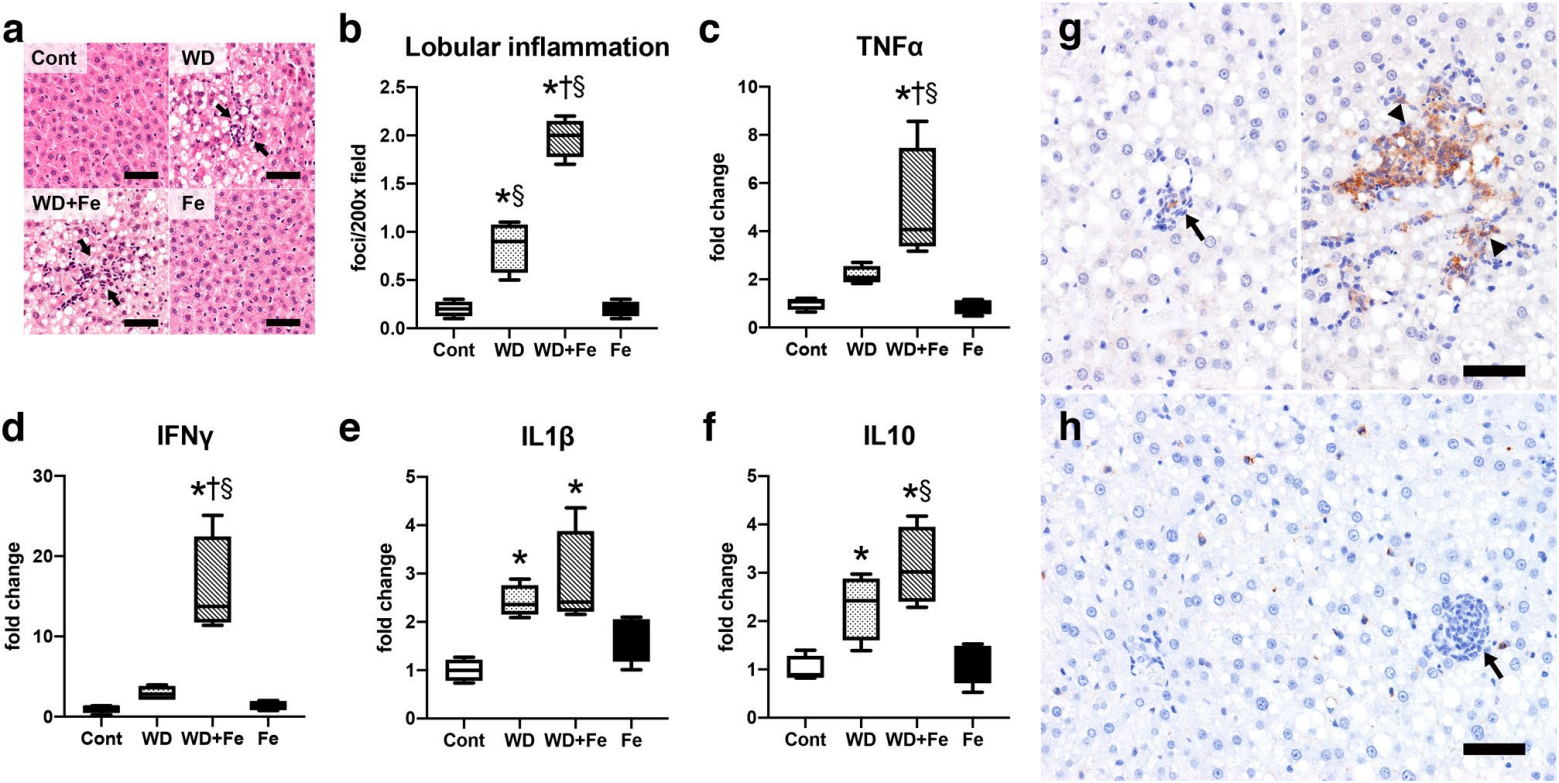
(**a**) Histological images representing microgranuloma (arrows) with mononuclear cell infiltrate in the fatty liver of WD and WD + Fe group. Inflammation is absent to minimal in Cont and Fe groups. (**b**) The number of inflammatory foci in the hepatic lobule at weeks 26. Hepatic expression of (**c**) TNFα, (**d**) IFNγ, (**e**) IL1β, and (**f**) IL10 mRNA at week 26. Data were normalized to 18S rRNA and expressed as fold change from control. Data are presented as box and whiskers (n = 4/group). *P < 0.05 versus Cont, ^†^*P* < 0.05 versus WD, and ^§^*P* < 0.05 versus Fe by one-way ANOVA followed by Tukey’s multiple comparison. Images of IHC for (**g**) iNOS and (**h**) CD206 in WD + Fe group at week 26. Arrows indicate microgranulomas while arrowheads indicate large inflammatory foci. Bar; 50 μm.

Histopathological examination revealed an increased number of inflammatory foci in the hepatic lobule (Fig. 6a, b) in WD (*P* = 0.0011 vs. Cont and *P* = 0.0011 vs. Fe) and WD + Fe (*P* < 0.0001 vs. Cont and *P* < 0.0001 vs. Fe) groups; the number is much higher in WD + Fe than in WD group (*P* < 0.0001). Hepatic expression of cytokine genes increased in WD (IL1β; *P* = 0.0254 vs. Cont, IL10; *P* = 0.038 vs. Cont) and/or WD + Fe (TNFα; *P* = 0.0035 vs. Cont and *P* = 0.0028 vs. Fe, IFNγ; *P* = 0.0001 vs. Cont and *P* = 0.0001 vs. Fe, IL1β; *P* = 0.0047 vs. Cont, IL10; *P* = 0.0013 vs. Cont and *P* = 0.0026 vs. Fe) groups (Fig. 6c–f); expression of TNFα and IFNγ was higher in WD + Fe than in WD group (TNFα; *P* = 0.0352, IFNγ; *P* = 0.0004). Tissue macrophages are functionally classified into classically-activated M1-type (with pro-inflammatory properties) and alternatively-activated M2-type (with anti-inflammatory or pro-resolving properties)^33^. Immunohistochemistry revealed that the inflammatory foci in the liver of WD and WD + Fe groups consisted of iNOS-positive M1 macrophages; the immunoreactivity tended to be more intense in large inflammatory foci (Fig. 6g, indicated by arrowheads) than in small foci (microgranuloma; Fig. 6g, arrow) and more intense in WD + Fe than in WD group (Supplementary Fig. S4a–d). CD206-positive M2 macrophages^33^ were sporadically seen in the sinusoid, with no direct relationship with inflammatory foci (Fig. 6h, arrow, Supplementary Fig. S4e–f). Expression of TNFα mRNA also increased in the visceral adipose tissue of WD + Fe group, compared with Cont and WD groups (Supplementary Fig. S5a). Adipose tissue expression of leptin decreased in WD and WD + Fe groups (Supplementary Fig. S5b). Expression of IL6, adiponectin, and genes involved in adipocyte metabolism (PPARγ, Pde3b, Srebf1, Cpt1a, Acaca, and Dgat1) did not differ significantly between groups (Supplementary Fig. S5c–j).

**Figure 7:**
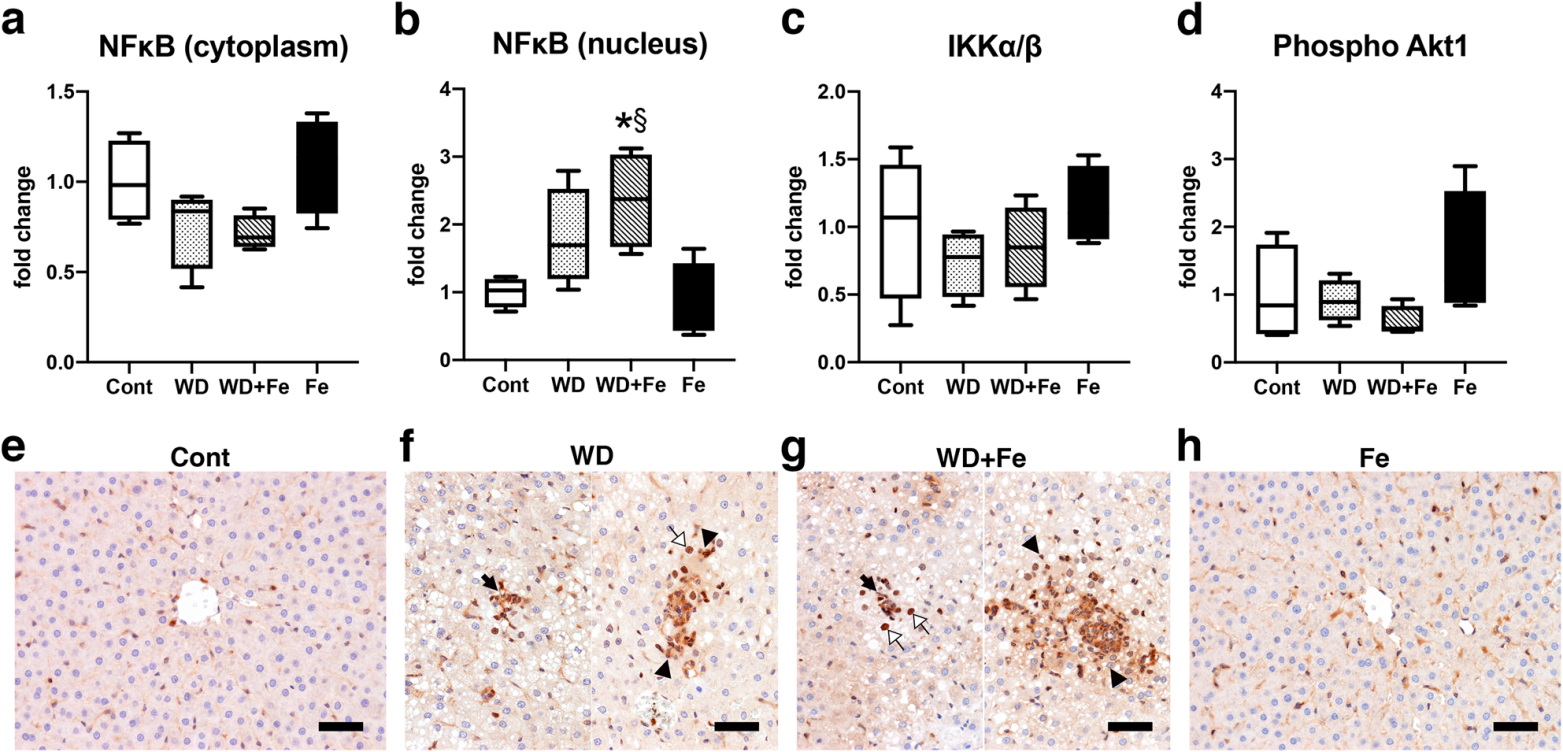
Hepatic expression of NFκB in the (**a**) cytoplasmic and (**b**) nuclear fractions, and (**c**) phospho IκB kinases and (**d**) phospho Akt in the whole liver tissue lysate at week 26. GAPDH and histone H2B were used for a loading control in the whole tissue/cytoplasmic and nuclear fraction, respectively. Data are expressed as fold change from control and are presented as box and whiskers (n = 4/group). *P < 0.05 versus Cont, ^§^*P* < 0.05 versus Fe by one-way ANOVA followed by Tukey’s multiple comparison. Images of NFκΒ (p65 or RelA) immunohistochemistry in Cont (**e**), WD (**f**), WD + Fe (**g**), and Fe (**h**) groups. Bar = 50 μm.

In order to clarify the mechanism for the increased hepatic inflammation by the dietary iron overload, nuclear factor kappa B (NFκB), a transcription factor involved in innate and adaptive immune responses as well as a series of pathological inflammation^34^, was targeted in this study. Nuclear translocation of NFκB activates transcription of downstream target genes such as TNFα^34^. NFκB level in the nuclear fraction of the liver increased in WD + Fe group (*P* = 0.03 vs. Cont and *P* = 0.0163 vs. Fe) (Fig. 7a), while cytoplasmic NFκB expression did not change significantly between all groups (Fig. 7b). Next, we focused on the upstream pathway of NFκB. IκB kinase (IΚK) complex, consisting of two kinases (IΚKα and IΚKβ), is the core regulator of NFκB. Activated IΚK phosphorylates and degrades IκB, the binding protein of NFκB, resulting in nuclear translocation of NFκB^34,35^. Phosphorylated-Akt (including Akt1 and Akt2) is also known as an NFκB activator via IΚKα phosphorylation^36^. In this study, there were no significant changes in hepatic expression of phosphorylated IΚK (Fig. 7c) and phosphorylated-Akt (Fig. 7d) between all groups in our model. Additionally, as mentioned above, hepatic expression of phosphorylated Akt2 rather decreased in the WD + Fe group (Fig. 5d). Immunoreactivity of NFκB was sporadically present in the nucleus of sinusoidal cells (probably Kupffer cells) of Cont and Fe groups (Fig. 7e, h). Moderate to intense immunoreactivity was seen in the nucleus and cytoplasm of inflammatory cells in the microgranulomas (Fig. 7f, g; black arrows) and large inflammatory foci (Fig. 7f, g; black arrowheads) in WD and WD + Fe groups; some hepatocytes around the inflammatory foci had nuclear immunoreactivity of NFκB (Fig. 7f, g; white arrows).

## Discussion

NAFLD develops in close relationship with MetS, particularly with obesity, insulin resistance, T2DM and dyslipidemia^4^. The Western diet feeding induced hepatic steatosis, hyperlipidemia, and partly-altered hepatic insulin pathway in the present rat model for NAFLD. This model also represents hepatic inflammation with upregulation of inflammatory cytokines and mild liver injury, indicative of the transitional phenotypes from NAFL to early NASH. Addition of dietary iron overload to the Western diet feeding resulted in hypertriglyceridemia, hypercholesterolemia, hepatic iron accumulation, increased hepatic lipid peroxidation, and exacerbation of hepatic inflammation with upregulation of M1-related cytokines and nuclear translocation of NFκB in the present WD + Fe model. Despite the presence of many evidences suggesting that excessive iron is involved in the pathology of NAFLD, the mechanism has not been fully elucidated since the disease condition is complicated by various factors such as insulin resistance, immune status, and oxidative stress^4,5^. Our results suggested that iron overload can alter metabolic condition and enhance hepatic inflammation in the fatty liver disease, shedding light on the pathological link between NAFLD and DIOS in humans.

DIOS is clinically defined on the basis of the following findings: the presence of MetS components, hyperferritinemia with normal transferrin saturation, and mild hepatic iron excess typically with sinusoidal accumulation^17–21^. However, the pathogenesis of DIOS is still debated. The present WD model has an increased serum iron with normal transferrin saturation, suggesting a normal capacity of serum transferrin to bind iron. Combination of dietary iron supplementation with Western diet feeding induces hepatic iron accumulation mainly along the sinusoid with hepatic hepcidin upregulation, as in human DIOS and NAFLD^18–20,37^. As shown in Table 1, MetS phenotypes are similar between the present WD + Fe model, human NAFLD and DIOS, suggesting a phenotypical relevance of this model to human DIOS and NAFLD with iron overload. Serum ferritin is commonly measured as a surrogate marker for body iron stores in human clinical practice, instead of direct measurement of tissue iron^38,39^. However, serum ferritin is independent of body iron stores in rats because it contains only a small amount of iron^40^. Thus, we evaluated hepatic iron content in our rat model when comparing body iron store conditions with those in humans. The average daily iron intake in Fe (485.2 mg/kg body weight/day) and WD + Fe (444.3 mg/kg body weight/day) groups is approximately 32 times and 30 times higher than in control rats (15.1 mg/kg body weight/day), respectively. With the body weight-based calculation, the average daily iron intake in Fe and WD + Fe groups is approximately 1777 times and 1940 times higher than in the average daily iron intake of male (0.25 mg/kg body weight/day), respectively^41^. Hepatic iron content in NAFLD patients with hyperferritinemia and/or abnormal serum iron is 980–5070 μg/g dry weight, which is normal to mild-moderate iron overload in the clinical iron overload evaluation scale^42–44^. In the present WD + Fe model, hepatic iron content can be calculated to be 858–1574 μg/g dry weight, suggesting normal to mild increase in the hepatic iron^45^. It is considered that our WD + Fe model has a similar iron status to human NAFLD patients with iron dysregulation^44^.

**Table 1:** Comparison of clinical features of NAFLD with hyperferritinemia, DIOS and this model.

Clinical findings	NAFLD with hyperferritinemia^18,20^	DIOS^18,20^	The present rat WD + Fe model
Body iron stores (serum ferritin or liver iron)	Increased	Increased	Increased
Transferrin saturation	Slightly increased^72^	Normal	Normal
Hepcidin	Increased^73,74^	Increased^75^	Increased
Hepatic iron stores	Mildly increased^76–78^	Mildly increased	Mildly increased
Hepatic iron distribution	Non-parenchymal or mixed	Non-parenchymal or mixed	Mainly non-parenchymal
MetS components	Present	Present	Present
Steatosis	Present	Present	Present
Insulin signal	Resistant^73,74^	Resistant	Partly altered (hepatic insulin signal)
Cytokines	Increased in serum (IL6, TNFα, MCP1)^79,80^	Not available	Upregulated in the liver (TNFα, IFNγ)

The limitation of the present WD + Fe models is that hepatic insulin resistance is not fully altered as in typical NAFLD and DIOS. Insulin resistance is a major part of the ‘multiple hits’ leading to the development and progression of NAFLD, together with lipotoxicity, oxidative stress, and inflammatory cascade activation^5^. Hepatic insulin resistance, defined as impaired suppression of glucose production by insulin in hepatocytes, is also an important component in metabolic disorders^30,46^. Hepatic insulin resistance develops before systemic insulin resistance, indicating that hepatic insulin resistance can initiate development of whole-body insulin resistance^47^. Hepatic insulin pathway was shown to be partly altered in the present WD and WD + Fe models; however, nuclear translocation of FOXO1 was unaffected with downregulation of glycogenesis genes. In the liver, gluconeogenesis can be suppressed by PDK1-dependent disassembly of the cAMP-response-element-binding protein complex as well as AKT2-FOXO1 pathway^48^. As for insulin receptor substrates, not only IRS1 but also IRS2 are involved in inhibition of hepatic glucose output^49^. It is considered that glycogenesis might be regulated via these alternative pathways in response to IRS1-Akt 2 suppression in this model. Fructose and glucose challenge in drinking water was selected in order to alter hepatic insulin cascade in this model. Fructose and/or glucose solution has been widely used to induce MetS phenotypes in rodent models since excessive intake of fructose and glucose is suggested as the main cause of the MetS development^47,50,51^. It would be effective to increase sugar content as much as previous studies showing hepatic or systemic insulin resistance^50,52^, for obtaining definite insulin resistance in our model. Another method to facilitate MetS in our model is to feed Western diet on well-established diabetes models, such as chemically-induced and genetically-modified models. The former includes a T2DM model with low-dose streptozotocin treatment^53^. However, it should be noted that streptozotocin-induced β-cell disorder is much more severe than natural pathogenesis^53,54^. The latter includes rodents with leptin-leptin receptor axis deficiency (*ob/ob* mouse, *db/db* mouse, Zucker fatty rat) and with spontaneous D2M (KK mouse); these animals develop insulin resistance with obesity^51,54^. The combination of diabetes induction with Western diet feeding could produce a better model that more clearly mimics human NAFLD and MetS.

In this study, dietary iron overload exacerbates Western diet-induced hepatic inflammation with upregulation of cytokines such as TNFα and IFNγ; similar findings were obtained in the previous studies with rodent NAFLD models^22,55^. Additionally, the exacerbation of hepatic inflammation in the present WD + Fe model was shown to be associated with intense iron accumulation in macrophages/Kupffer cells and increased nuclear translocation of NFκB. Nuclear translocation of NFκB was especially remarkable in the inflammatory foci of the WD + Fe model. TNFα is associated with obesity and insulin resistance, and is also known as an activator of NFκB-driven inflammation by binding to TNFR1^34,56,57^. Expression of hepatic and adipose TNFα and hepatic TNFα receptor increases in NASH obese patients compared with that in non-NASH obese^58^. Similarly, TNFα expression is upregulated both in the liver and visceral adipose tissue in the present WD + Fe model, suggesting that TNFα produced from the liver and visceral adipose tissue could contribute to the exacerbation of hepatic inflammation. Increased NFκB activity is also associated with the development of MetS components such as steatosis, hepatic insulin resistance, and inflammation in rodents^5,57,59^. NFκB activation is also proven in the liver biopsies from NASH patients^60,61^. Additionally, several studies suggest that excessive iron directly induces NFκB activation and TNFα upregulation via IΚK in cultured Kupffer cells^62,63^. TNFα-NFκB interaction might promote hepatic inflammation in the present WD + Fe model. IFNγ originates largely from T-helper cells and is mainly associated with the Th1 type immune responses^64^. Although data are limited on the role of Th1 cells in human NAFLD, increased numbers of IFNγ-producing T cells were observed in NASH patients^65^. IFNγ also can activate M1 macrophages that secrete pro-inflammatory cytokines such as TNFα and IL1β^33,66^. In present WD + Fe model, the mononuclear leukocytes in the inflammatory foci were strongly positive for iNOS, while they are negative for CD206, suggesting that M1 macrophages mainly consist of the inflammatory foci. Iron-induced Th1/M1 immune response might also contribute to the enhanced hepatic inflammation in the present WD + Fe model.

Dyslipidemia is one of the main clinical features of NAFLD as well as MetS with its high prevalence (20% to 80% of NAFLD patients) and it has been noted as a risk factor for cardiovascular disease^67^. These evidences would support the usefulness of this model as it represents the important phenotype of human NAFLD with multiple MetS components. Additionally, dietary iron overload increases serum triglyceride and total cholesterol, but not hepatic triglyceride in the present WD + Fe model. Graham et al*.* demonstrated that hepatic iron loading increases liver cholesterol synthesis in mice^68^. In addition, activation of NFκB signaling pathway in hepatocytes, as seen in the present WD + Fe model, is shown to promote lipogenesis including cholesterol synthesis^69^. These findings suggest that hepatic iron overload can alter lipid metabolism, particularity cholesterol metabolism.

Oxidative stress is regarded as one of the “multiple hits” of NAFLD and is thought to be associated with activation of innate immune signaling during development of NAFLD via activation of transcription factors such as interferon regulatory factors and NFκB^27,28,70^. Iron overload can induce tissue injury via generation of hydroxyl radical, a highly reactive oxygen species, by the Fenton reaction^9^. However, the increase in hepatic MDA content, a marker for lipid peroxidation^28^, was mild (less than twofold) in the present WD + Fe model, without significant changes in serum transaminases or hepatic GSH/GSSG ratio, an indicator of oxidative stress-antioxidant balance^27^. Additionally, hepatic iron accumulation was mild to moderate in the present WD + Fe models as in patients with NAFLD with hyperferritinemia or DIOS^19,20^. These findings suggest that non-hepatotoxic amount of iron accumulation can promote dyslipidemia and hepatic inflammation via macrophage/Kupffer cell activation, rather than induce direct liver injury in metabolic liver diseases.

In conclusion, our results demonstrated that the new NAFLD model with hepatic iron overload shares many features of human NAFLD with hyperferritinemia and DIOS. Addition of dietary iron overload to Western diet-induced fatty liver disease exacerbates hepatic inflammation with activation of NFκB and alters lipid metabolism, suggesting an important role of iron in the development and progression of NAFLD as one of the "multiple hits” factors. Further improvement of the experimental model would elucidate more clearly the crosslink between human NAFLD and DIOS.

## Materials and methods

### Animals

The experiment conducted in this study is reported in accordance with the ARRIVE guidelines (https://arriveguidelines.org). Ten-week-old male F344/DuCrlCrlj rats (Charles River Laboratories Japan, Yokohama, Japan) were divided into control (Con), Western diet (WD), and Western diet + high-iron (WD + Fe), and high-iron (Fe) groups (n = 4 in each group); average of the body weight is similar between groups at the beginning of the experiment. A sample size of four per group was determined in terms of reduction (minimizing the number of animals) and experimental accuracy (minimizing the influence of individual variations). Two or three rats per cage were maintained in a room with controlled temperature and 12-h light–dark cycle. Food and water were provided ad libitum. Rats were fed diets shown in Supplemental Table S1 for 26 weeks. High iron diet was prepared by blending iron citrate (FeC_6_H_5_O_7_・5H_2_O) at a concentration of 6%. Body weight, food intake, and water intake were measured once a week. After 26-week feeding, rats were euthanized under deep isoflurane anesthesia, and the whole blood, liver, subcutaneous (inguinal) and visceral (peri-epidydimal) fat tissues, spleen, pancreas, cecal content, heart, kidneys, and lungs were collected. All rats were included in the analyses described below without any exclusion. Confounders were not controlled in this study; however, the influence of the order of treatments and measurements is considered minimal as far as observing the data obtained. Two of the authors (S.F and T.I.) were aware of the group allocation at the different stages of the experiments. All experiments were approved by the Animal Care and Use Committee at Osaka Prefecture University (code nos. 29–184 and 30–71) and were performed according to the Guidelines for Animal Experimentation of Osaka Metropolitan University.

### Biochemical analyses

Blood was collected from the abdominal aorta and was left for one hour at room temperature. Serum was separated by centrifugation (3000 rpm, 10 min). Biochemical analyses were performed in SRL Inc. (Tokyo, Japan).

### Histopathology

The left lateral lobe and caudate of the liver were fixed in 10% neutral-buffered formalin, embedded in paraffin, cut at 5 µm, and stained with hematoxylin and eosin (HE) for histopathological examination. The number of inflammatory foci was counted. Ten 200 × fields of left lateral lobe sections were evaluated from each animal. The data were presented as the number of inflammatory foci per 200 × field.

### Hepatic triglyceride assay

To examine triglyceride content in the liver, hepatic triglyceride assay was performed with Triglyceride E-test Wako kit (Wako Pure Chemical Industries, Tokyo, Japan) according to the manufacturer’s instruction.

### Immunohistochemistry

Sections from the left lateral lobe and caudate of the liver were subjected to immunohistochemistry (IHC) with primary antibodies as listed in Supplemental Table S2. After dewaxing and antigen retrieval, tissue sections were immunostained in a Histostainer system (Nichirei Biosciences, Tokyo, Japan) as described previously^71^. Briefly, sections were treated with 5% skimmed milk in phosphate buffered saline (PBS) for 10 min, with each primary antibody at room temperature for 1 h, with 3% H_2_O_2_ in PBS for 15 min, and with horseradish peroxidase-conjugated secondary antibody (Histofine Simple Stain MAX PO; Nichirei Biosciences) at room temperature for 30 min. Positive reactions were visualized with 3,3′-diaminobenzidine (DAB substrate kit; Nichirei Biosciences). After immunohistochemistry, sections were stained with Perls solution for detection of tissue iron.

### Serum insulin assay

Serum insulin levels were analyzed with LBIS Rat Insulin ELISA Kit (Shibayagi Co., Gunma, Japan) according to the manufacture's instruction.

### Malondialdehyde (MDA) assay

To examine lipid peroxidation change, hepatic MDA contents were analyzed by thiobarbituric acid reactive substances (TBARS) method using an MDA Assay Kit (Northernwest Life Science Specialities, Vancouber, Canada) as described previously^71^.

### Glutathione-S–S-glutathione/glutathione-SH (GSSG/GSH) quantification

To examine hepatic antioxidant activity, hepatic GSSG/GSH ratio was analyzed using a GSSG/GSH Quantification Kit (Dojindo, Kumamoto, Japan) according to the manufacturer's instructions.

### RT-PCR

Real-time RT-PCR was performed to examine expression patterns of major cytokine genes and genes related with iron metabolism as described previously^71^. Liver samples from the right medial lobe, epidydimal fat tissue, and inguinal fat tissue were immersed in RNA later regent (Qiagen, Hilden, Germany) and stored at − 80 °C before use. Total RNA was extracted using an SV Total RNA Isolation System (Promega, WI, USA). Two-point-five microgram of total RNA was reverse-transcribed to cDNA by SuperScript VILO cDNA synthesis kit (Invitrogen, Carlsbad, CA, USA). Real-time PCR was performed with TaqMan gene expression assays (Life Technologies) in a PikoReal Real-Time 96 PCR System (Thermo Scientific, Massachusetts, USA). Details of probes are listed in Supplemental Table S3. Eukaryotic 18sRNA and βactin was used as reference genes. The data were calculated with the 2^−ΔΔCT^ method.

### Western blot

Whole tissue homogenates, from the right medial lobe were prepared as described previously^71^. For preparation of cytoplasmic/nuclear fractions, the liver samples from the right medial lobe were homogenized in a buffer containing 10 mM HEPES/KOH (pH 7.5), 10 mM NaCl, 3 mM MgCl, 0.5% NP-40, 1 mM PMSF and proteinase inhibitor cocktail. After centrifugation at 3100×*g* for 5 min, the supernatant was collected as cytoplasmic fraction. The precipitate was mixed with 200 μL of another buffer containing 10 mM HEPES/KOH (pH 7.5), 25 mM NaCl, 3 mM MgCl, 300 mM sucrose, 1 mM PMSF and proteinase inhibitor cocktail and centrifugated at 3100×*g* for 5 min. After treatment of 10 mg/mL DNase I and sonication on ice, the sample was collected as nuclear fraction. Protein concentration was determined by an absorption spectrometer using Bio-Rad Protein Assay (Bio-Rad Laboratories, CA, USA). Samples were separated on 5–20% gradient polyacrylamide gels and transferred to polyvinylidene difluoride (PVDF) membranes (Bio-Rad Laboratories). Membranes were cut into several pieces by size prior to incubation with primary antibodies and were then incubated overnight at 4 °C with primary antibodies as listed in Supplemental Table S4, followed by an incubation with peroxidase-conjugated secondary antibody (Histofine Simple Stain MAX PO; Nichirei Biosciences) for 30 min. Signals were visualized with ECL prime (GE Healthcare, Little Chalfont, UK), and quantified with a luminescent image analyzer (LAS-4000; GE Healthcare). The band images analyzed are shown in Supplementary Fig. S6a–m.

### Statistical analysis

Data are presented as mean ± SD. Statistical analyses were performed using one-way ANOVA followed by Tukey–Kramer's test by a Prism software (ver. 9.4.1.; GraphPad, San Diego, CA, USA; https://www.graphpad.com/scientific-software/prism/). A value of *P* < 0.05 was considered statistically significant.

## Supplementary Information

Supplementary Information.

## Data Availability

The datasets used and/or analyzed during the current study are available from the corresponding author on reasonable request.
