# Supplementary material for: Dietary iron overload enhances Western diet induced hepatic inflammation and alters lipid metabolism in rats sharing similarity with human DIOS
Source: Sci Rep. 2022 Dec 10;12:21414. doi: 10.1038/s41598-022-25838-3 (PMC9741655; doi:10.1038/s41598-022-25838-3)
Supplement: Supplementary file 1 — Supplementary Information. [file 41598_2022_25838_MOESM1_ESM.pptx]

## Slide 1
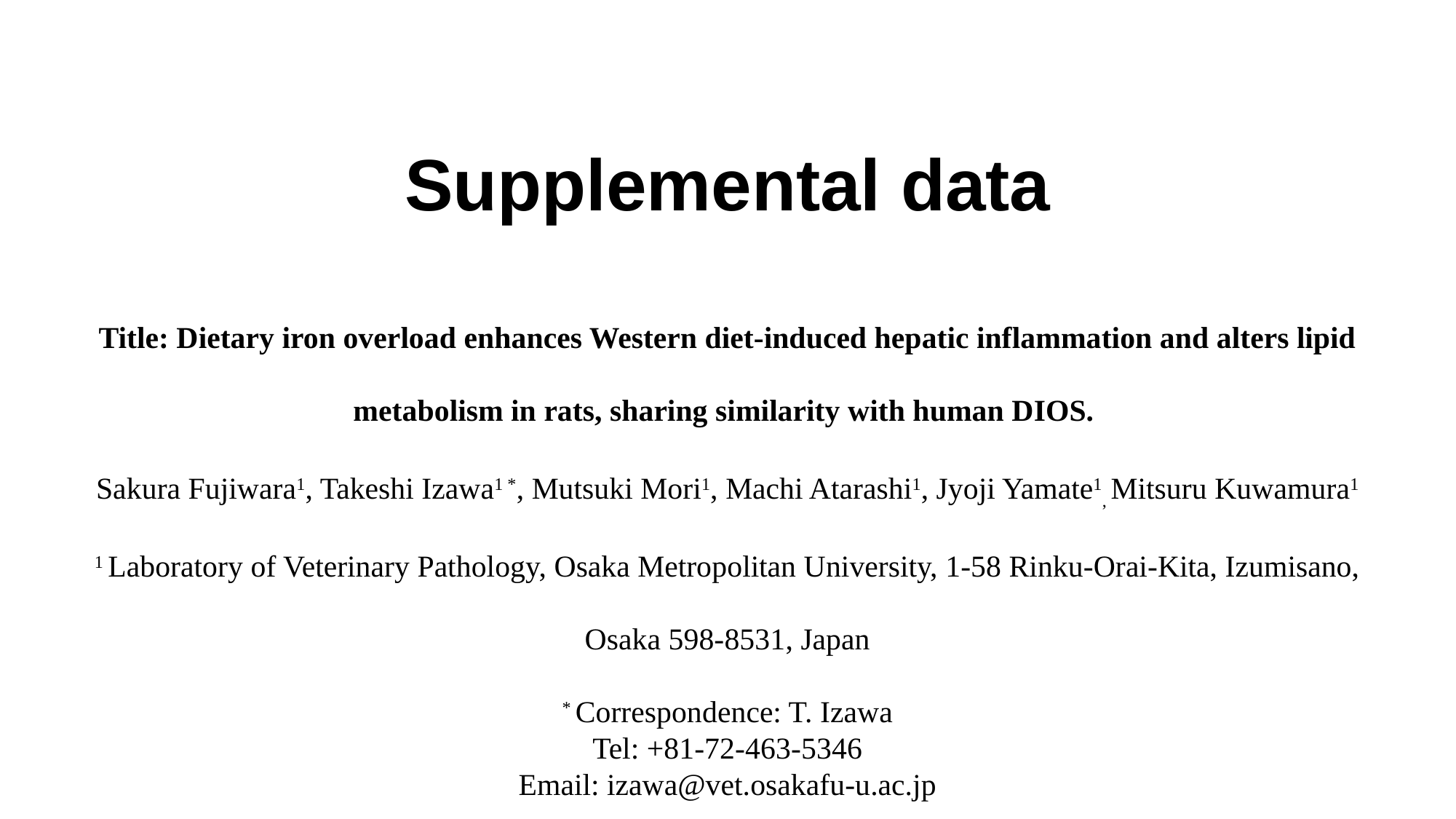

Supplemental data
Title: Dietary iron overload enhances Western diet-induced hepatic inflammation and alters lipid metabolism in rats, sharing similarity with human DIOS.
Sakura Fujiwara1, Takeshi Izawa1 *, Mutsuki Mori1, Machi Atarashi1, Jyoji Yamate1, Mitsuru Kuwamura1
1 Laboratory of Veterinary Pathology, Osaka Metropolitan University, 1-58 Rinku-Orai-Kita, Izumisano, Osaka 598-8531, Japan
* Correspondence: T. Izawa
Tel: +81-72-463-5346
Email: izawa@vet.osakafu-u.ac.jp

## Slide 2
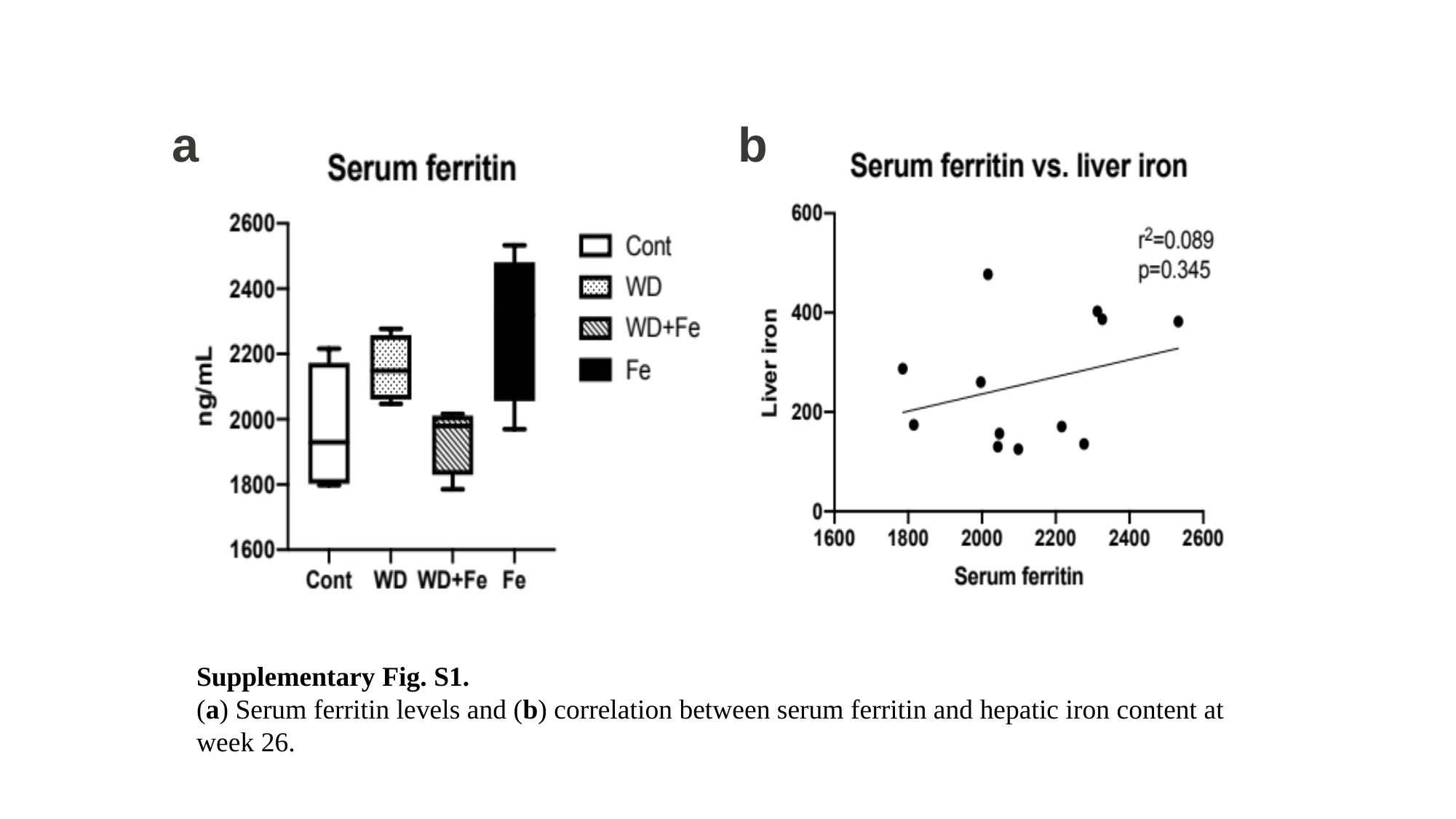

b
a
Supplementary Fig. S1.
(a) Serum ferritin levels and (b) correlation between serum ferritin and hepatic iron content at week 26.

## Slide 3
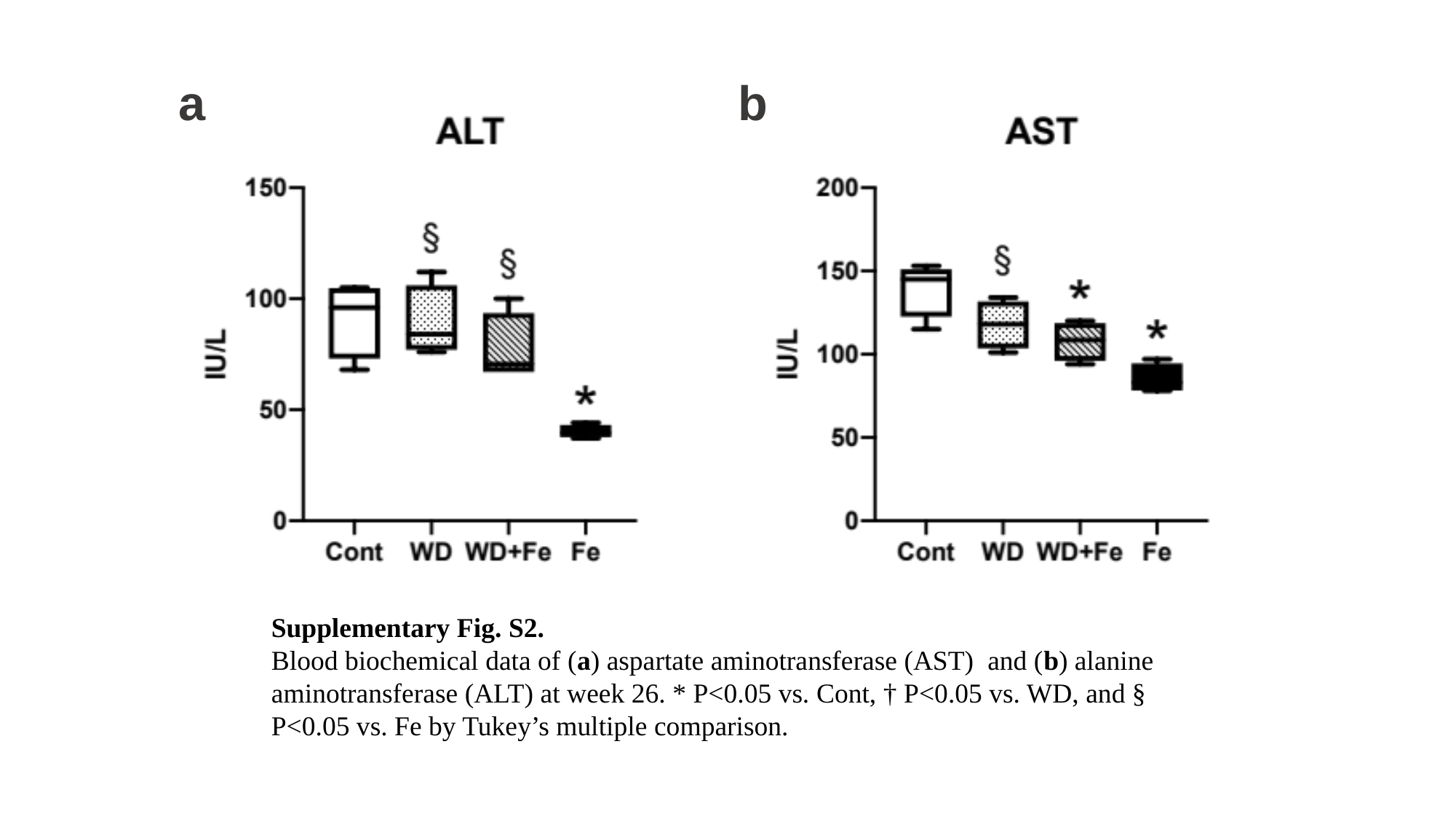

a
b
Supplementary Fig. S2.
Blood biochemical data of (a) aspartate aminotransferase (AST) and (b) alanine aminotransferase (ALT) at week 26. * P<0.05 vs. Cont, † P<0.05 vs. WD, and § P<0.05 vs. Fe by Tukey’s multiple comparison.

## Slide 4
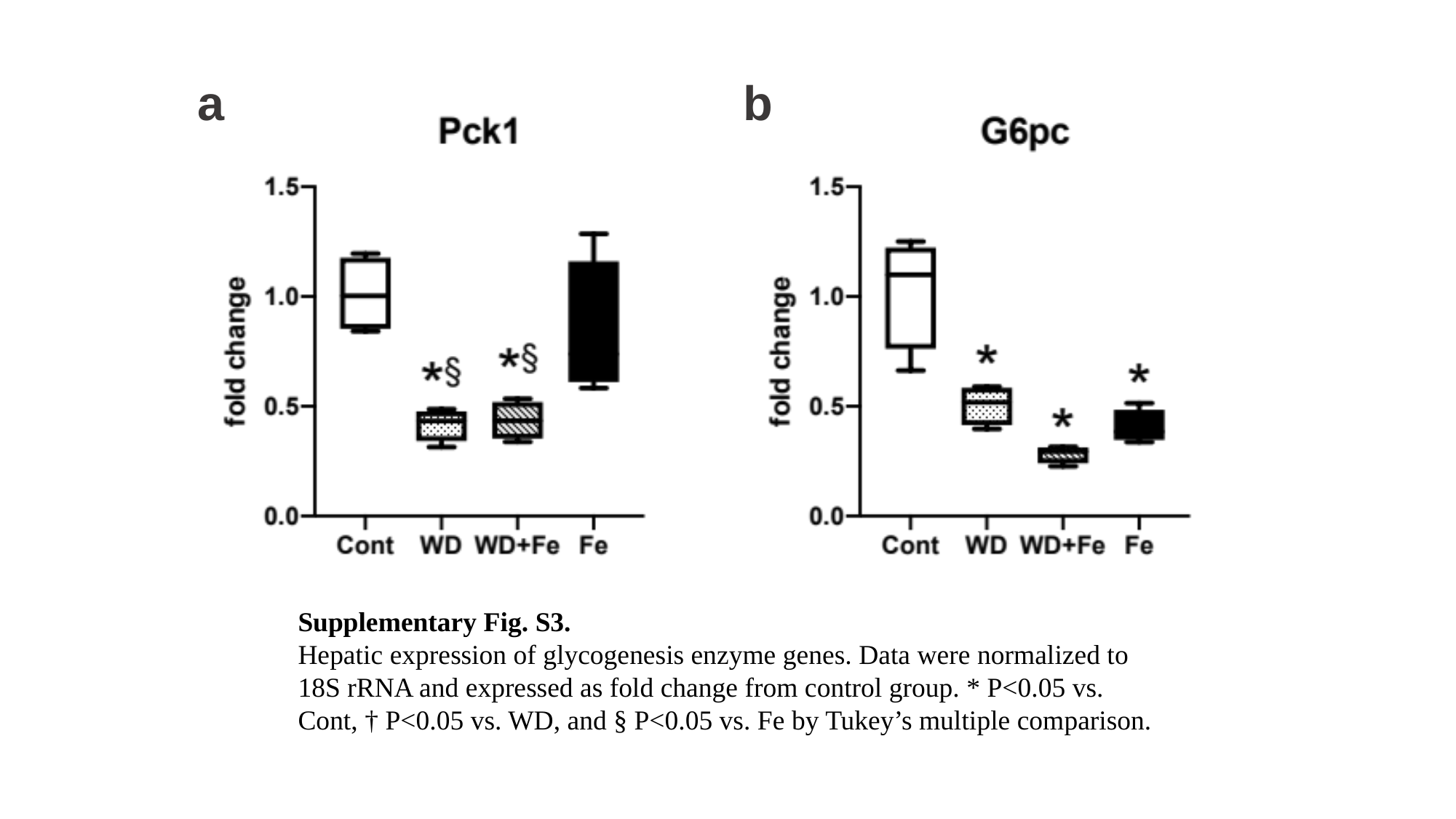

a
b
Supplementary Fig. S3.
Hepatic expression of glycogenesis enzyme genes. Data were normalized to 18S rRNA and expressed as fold change from control group. * P<0.05 vs. Cont, † P<0.05 vs. WD, and § P<0.05 vs. Fe by Tukey’s multiple comparison.

## Slide 5
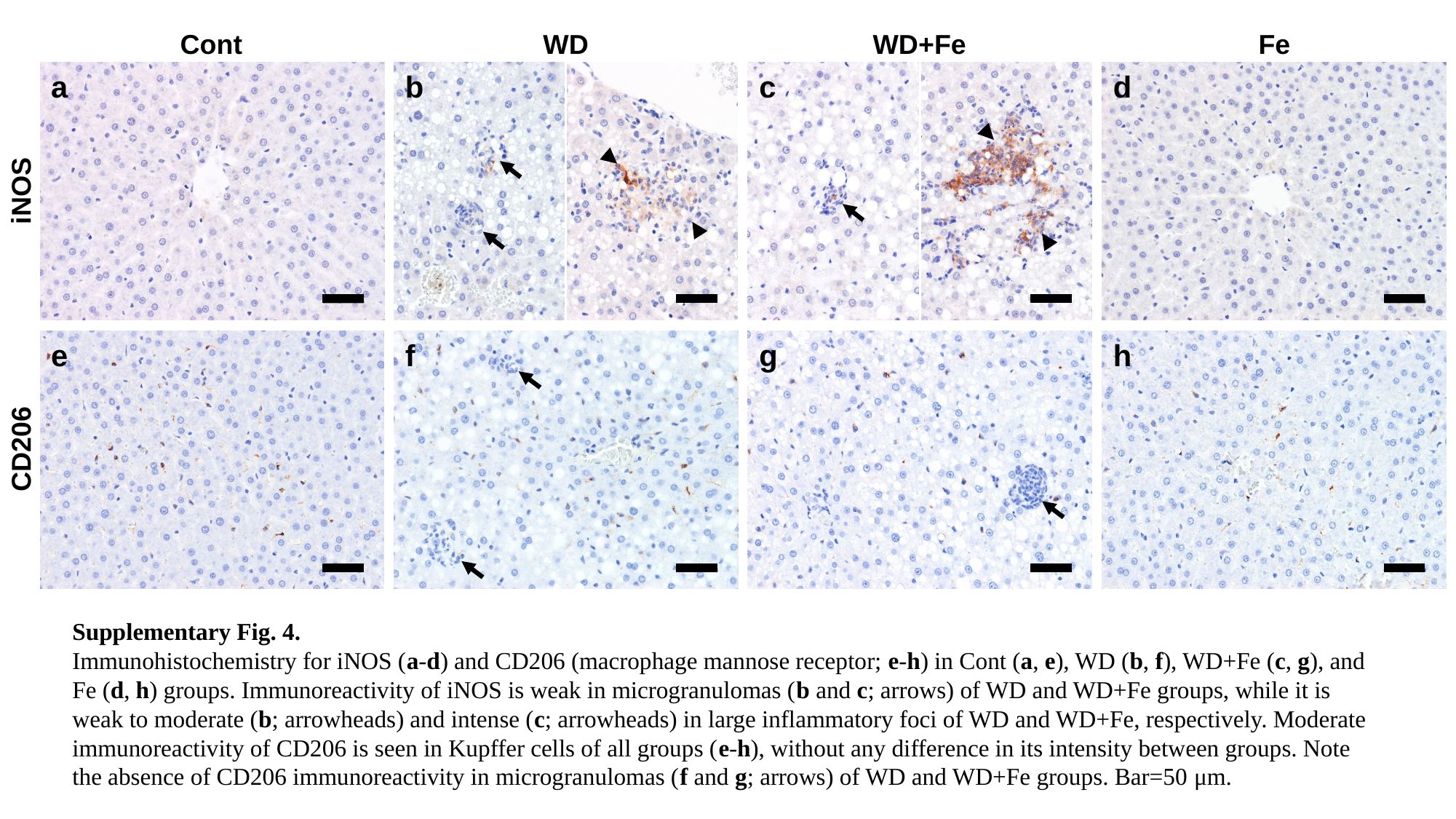

WD
WD+Fe
Fe
Cont
c
d
a
b
iNOS
g
h
e
f
CD206
Supplementary Fig. 4.
Immunohistochemistry for iNOS (a-d) and CD206 (macrophage mannose receptor; e-h) in Cont (a, e), WD (b, f), WD+Fe (c, g), and Fe (d, h) groups. Immunoreactivity of iNOS is weak in microgranulomas (b and c; arrows) of WD and WD+Fe groups, while it is weak to moderate (b; arrowheads) and intense (c; arrowheads) in large inflammatory foci of WD and WD+Fe, respectively. Moderate immunoreactivity of CD206 is seen in Kupffer cells of all groups (e-h), without any difference in its intensity between groups. Note the absence of CD206 immunoreactivity in microgranulomas (f and g; arrows) of WD and WD+Fe groups. Bar=50 μm.

## Slide 6
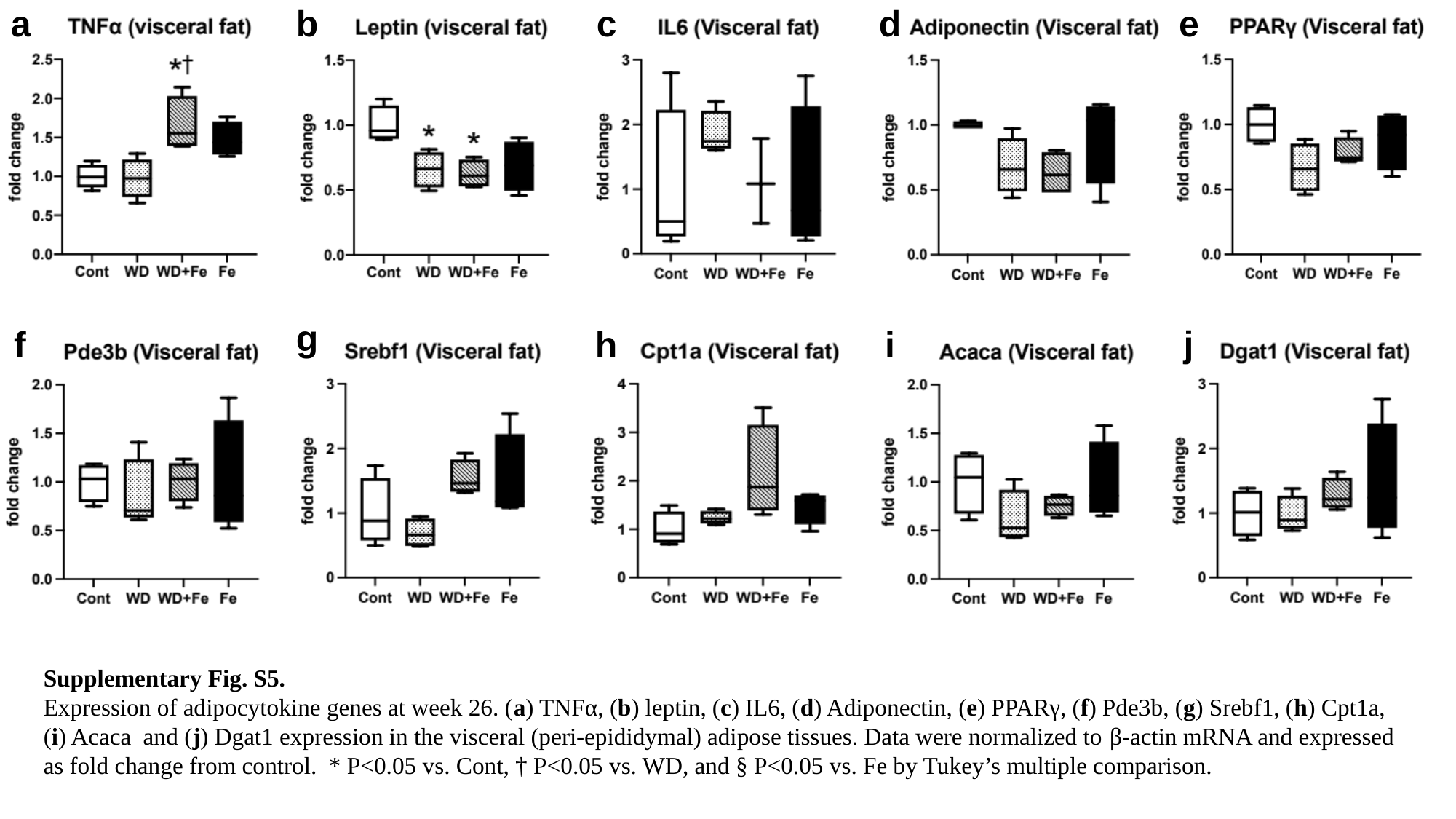

a
b
c
d
e
g
j
i
f
h
Supplementary Fig. S5.
Expression of adipocytokine genes at week 26. (a) TNFα, (b) leptin, (c) IL6, (d) Adiponectin, (e) PPARγ, (f) Pde3b, (g) Srebf1, (h) Cpt1a, (i) Acaca and (j) Dgat1 expression in the visceral (peri-epididymal) adipose tissues. Data were normalized to β-actin mRNA and expressed as fold change from control. * P<0.05 vs. Cont, † P<0.05 vs. WD, and § P<0.05 vs. Fe by Tukey’s multiple comparison.

## Slide 7
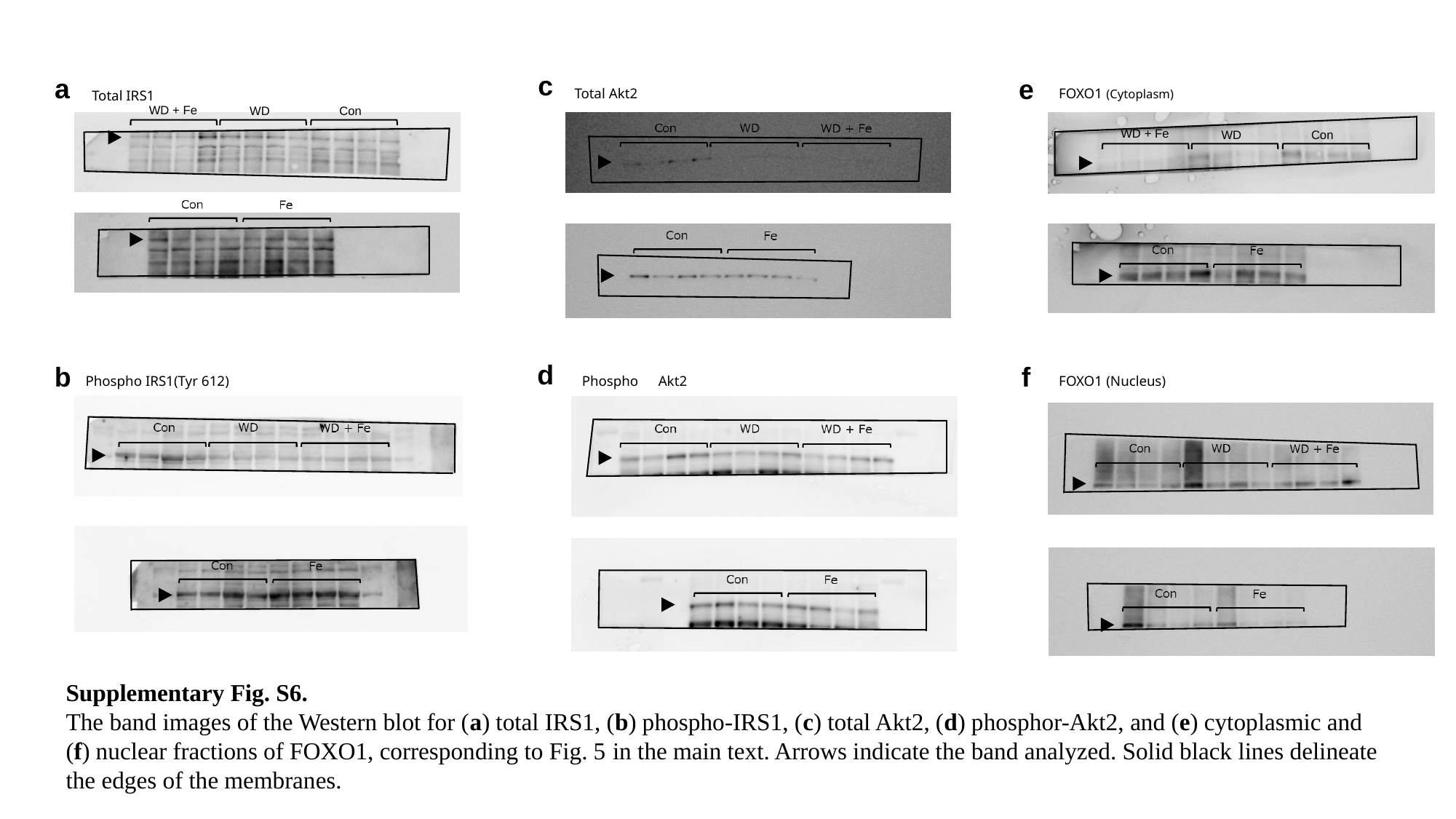

c
a
e
Total Akt2
FOXO1 (Cytoplasm)
Total IRS1
WD + Fe
WD
Con
WD + Fe
WD
Con
d
f
b
Phospho IRS1(Tyr 612)
Phospho　Akt2
FOXO1 (Nucleus)
Supplementary Fig. S6.
The band images of the Western blot for (a) total IRS1, (b) phospho-IRS1, (c) total Akt2, (d) phosphor-Akt2, and (e) cytoplasmic and (f) nuclear fractions of FOXO1, corresponding to Fig. 5 in the main text. Arrows indicate the band analyzed. Solid black lines delineate the edges of the membranes.

## Slide 8
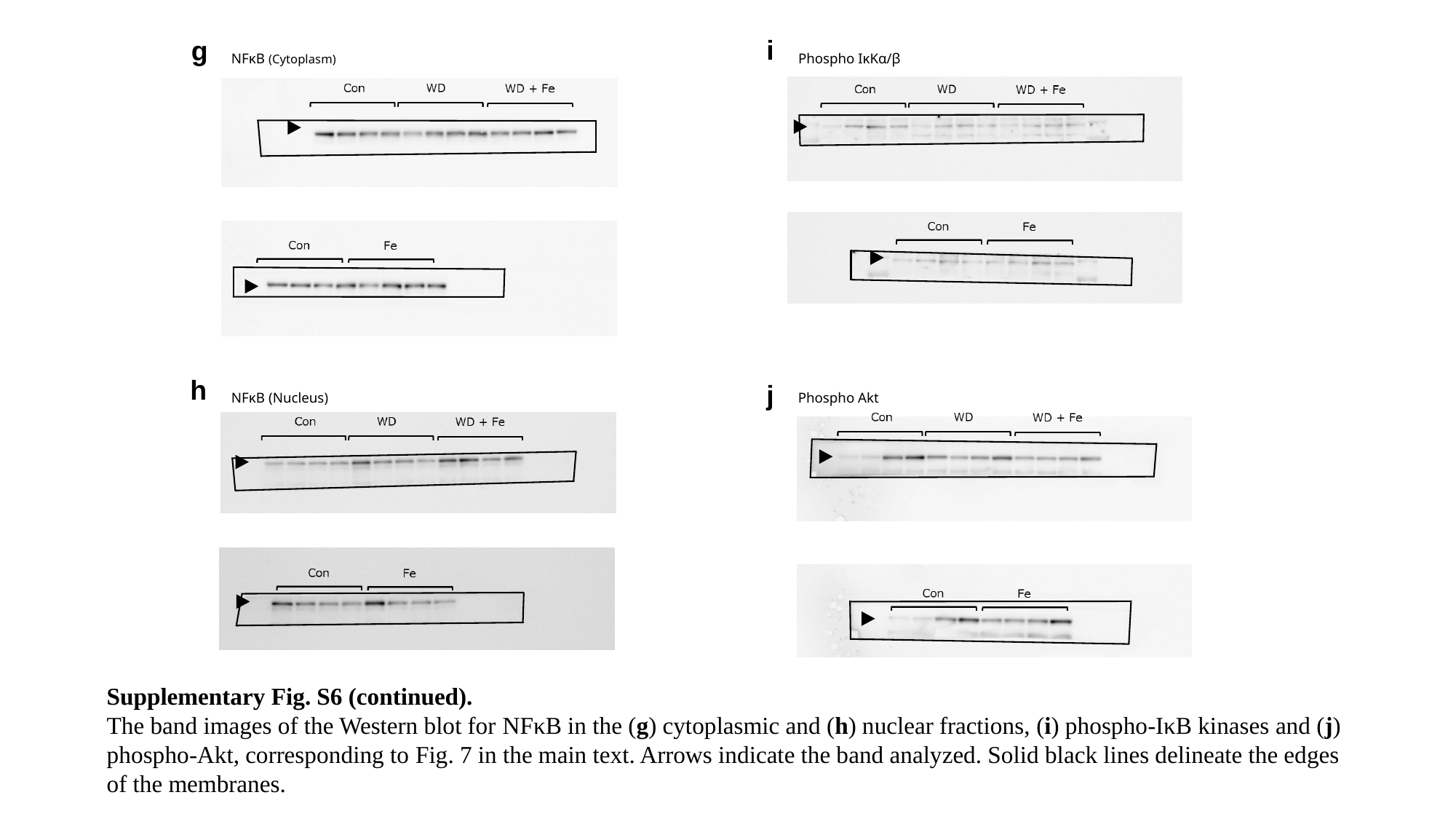

i
g
NFκB (Cytoplasm)
NFκB (Nucleus)
Phospho IκKα/β
h
j
Phospho Akt
Supplementary Fig. S6 (continued).
The band images of the Western blot for NFκB in the (g) cytoplasmic and (h) nuclear fractions, (i) phospho-IκB kinases and (j) phospho-Akt, corresponding to Fig. 7 in the main text. Arrows indicate the band analyzed. Solid black lines delineate the edges of the membranes.

## Slide 9
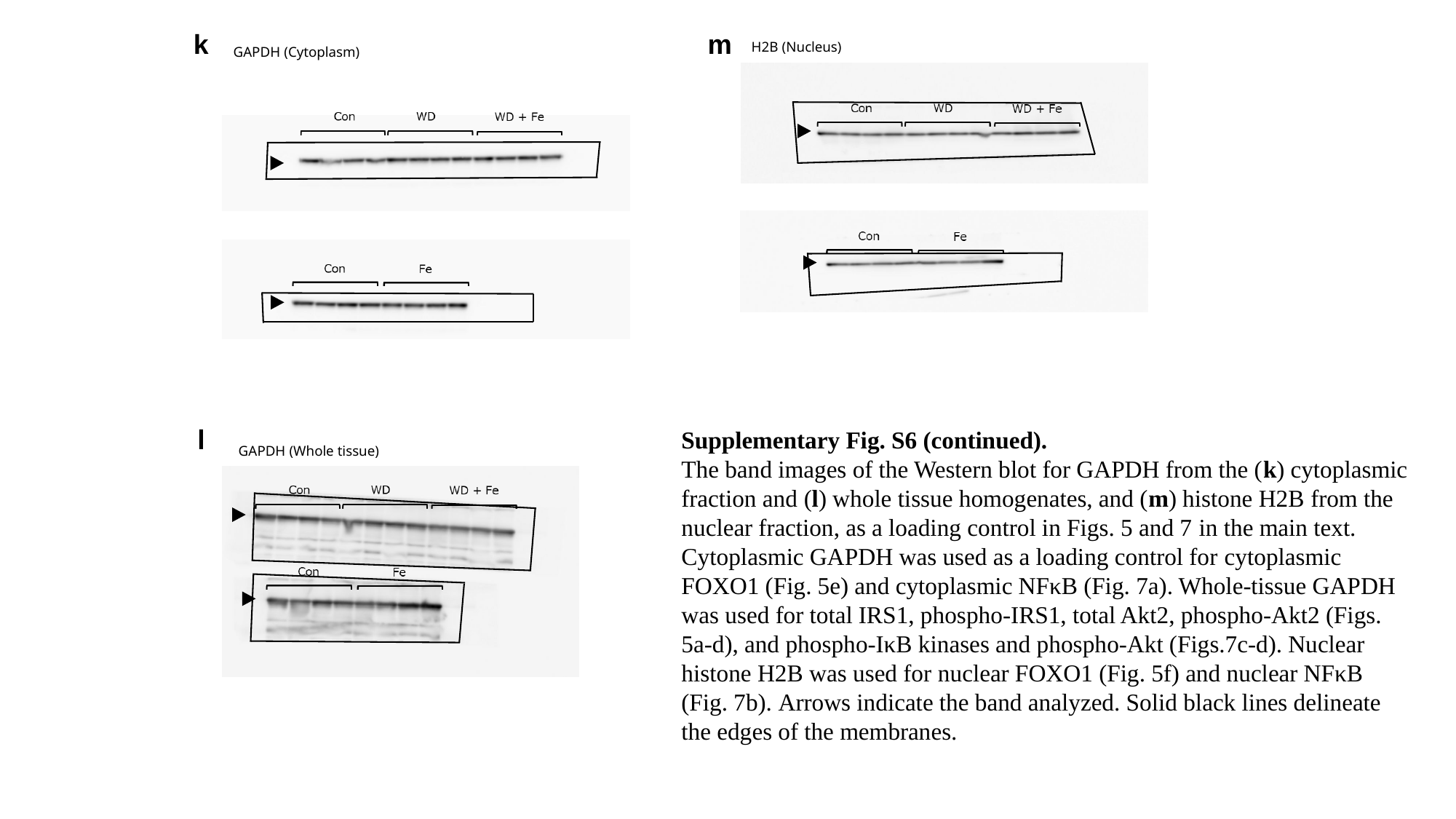

k
m
H2B (Nucleus)
GAPDH (Cytoplasm)
l
Supplementary Fig. S6 (continued).
The band images of the Western blot for GAPDH from the (k) cytoplasmic fraction and (l) whole tissue homogenates, and (m) histone H2B from the nuclear fraction, as a loading control in Figs. 5 and 7 in the main text. Cytoplasmic GAPDH was used as a loading control for cytoplasmic FOXO1 (Fig. 5e) and cytoplasmic NFκB (Fig. 7a). Whole-tissue GAPDH was used for total IRS1, phospho-IRS1, total Akt2, phospho-Akt2 (Figs. 5a-d), and phospho-IκB kinases and phospho-Akt (Figs.7c-d). Nuclear histone H2B was used for nuclear FOXO1 (Fig. 5f) and nuclear NFκB (Fig. 7b). Arrows indicate the band analyzed. Solid black lines delineate the edges of the membranes.
GAPDH (Whole tissue)

## Slide 10
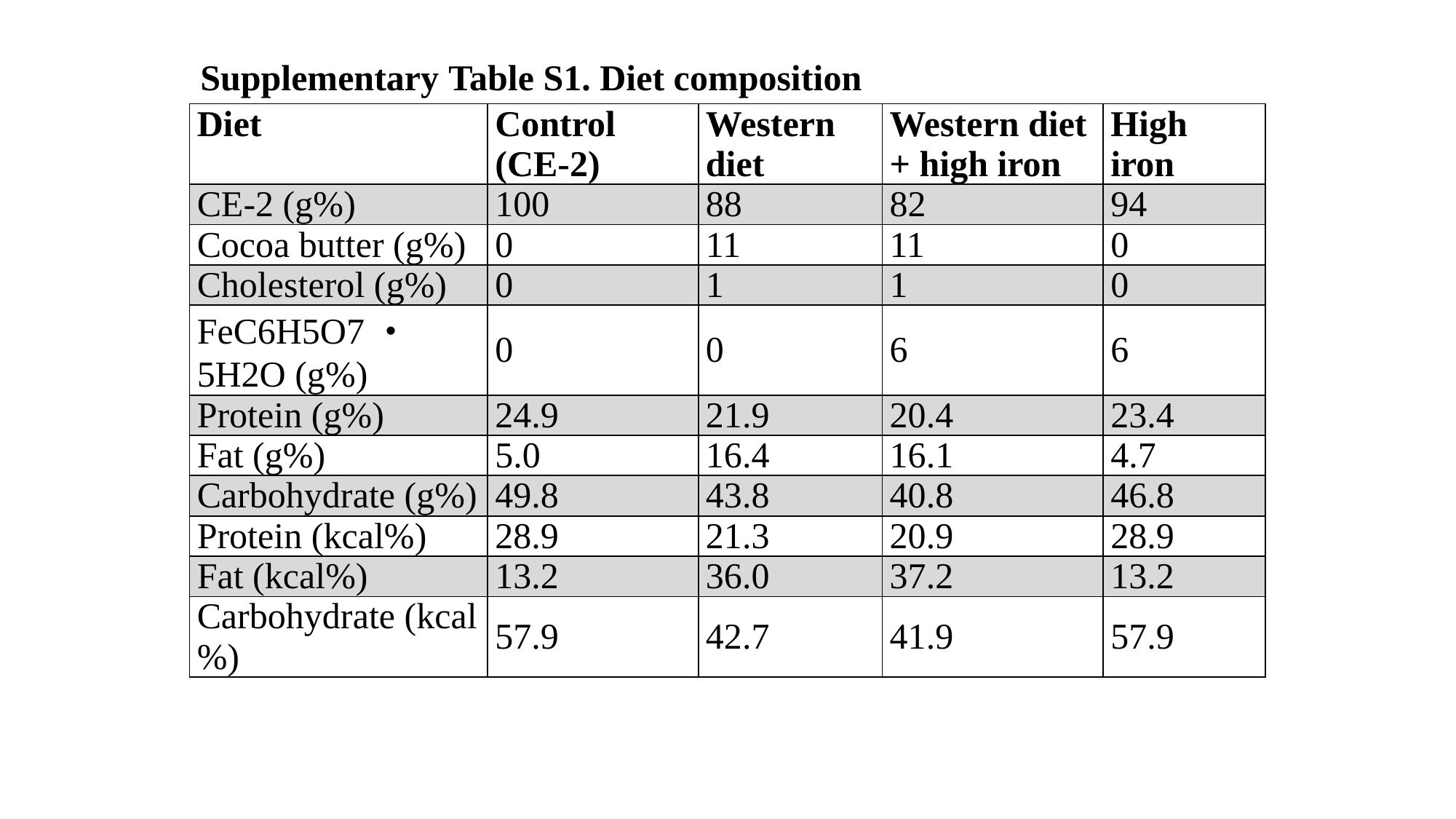

Supplementary Table S1. Diet composition
| Diet | Control (CE-2) | Western diet | Western diet + high iron | High iron |
| --- | --- | --- | --- | --- |
| CE-2 (g%) | 100 | 88 | 82 | 94 |
| Cocoa butter (g%) | 0 | 11 | 11 | 0 |
| Cholesterol (g%) | 0 | 1 | 1 | 0 |
| FeC6H5O7・5H2O (g%) | 0 | 0 | 6 | 6 |
| Protein (g%) | 24.9 | 21.9 | 20.4 | 23.4 |
| Fat (g%) | 5.0 | 16.4 | 16.1 | 4.7 |
| Carbohydrate (g%) | 49.8 | 43.8 | 40.8 | 46.8 |
| Protein (kcal%) | 28.9 | 21.3 | 20.9 | 28.9 |
| Fat (kcal%) | 13.2 | 36.0 | 37.2 | 13.2 |
| Carbohydrate (kcal%) | 57.9 | 42.7 | 41.9 | 57.9 |

## Slide 11
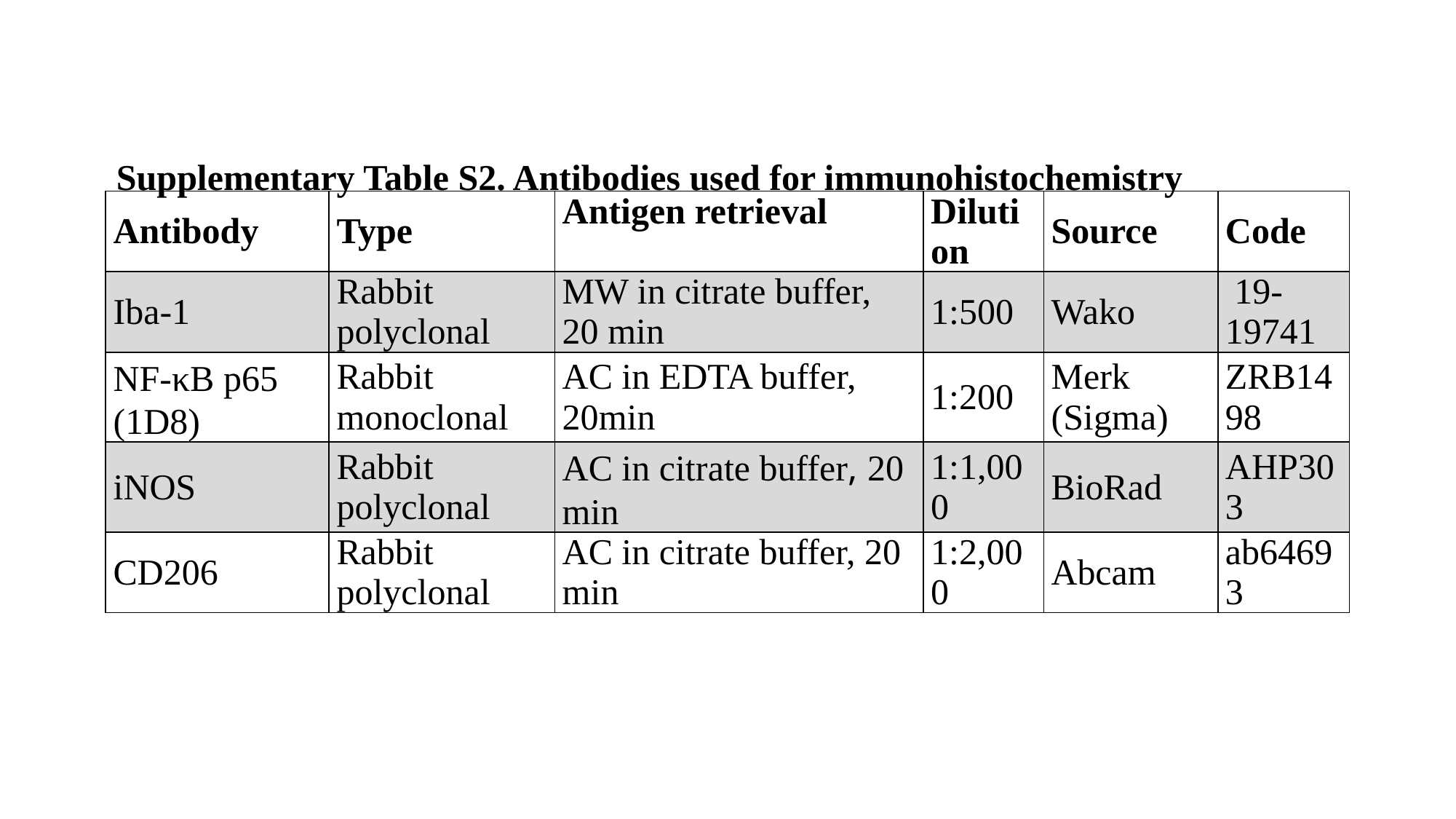

Supplementary Table S2. Antibodies used for immunohistochemistry
| Antibody | Type | Antigen retrieval | Dilution | Source | Code |
| --- | --- | --- | --- | --- | --- |
| Iba-1 | Rabbit polyclonal | MW in citrate buffer, 20 min | 1:500 | Wako | 19-19741 |
| NF-κB p65 (1D8) | Rabbit monoclonal | AC in EDTA buffer, 20min | 1:200 | Merk (Sigma) | ZRB1498 |
| iNOS | Rabbit polyclonal | AC in citrate buffer, 20 min | 1:1,000 | BioRad | AHP303 |
| CD206 | Rabbit polyclonal | AC in citrate buffer, 20 min | 1:2,000 | Abcam | ab64693 |

## Slide 12
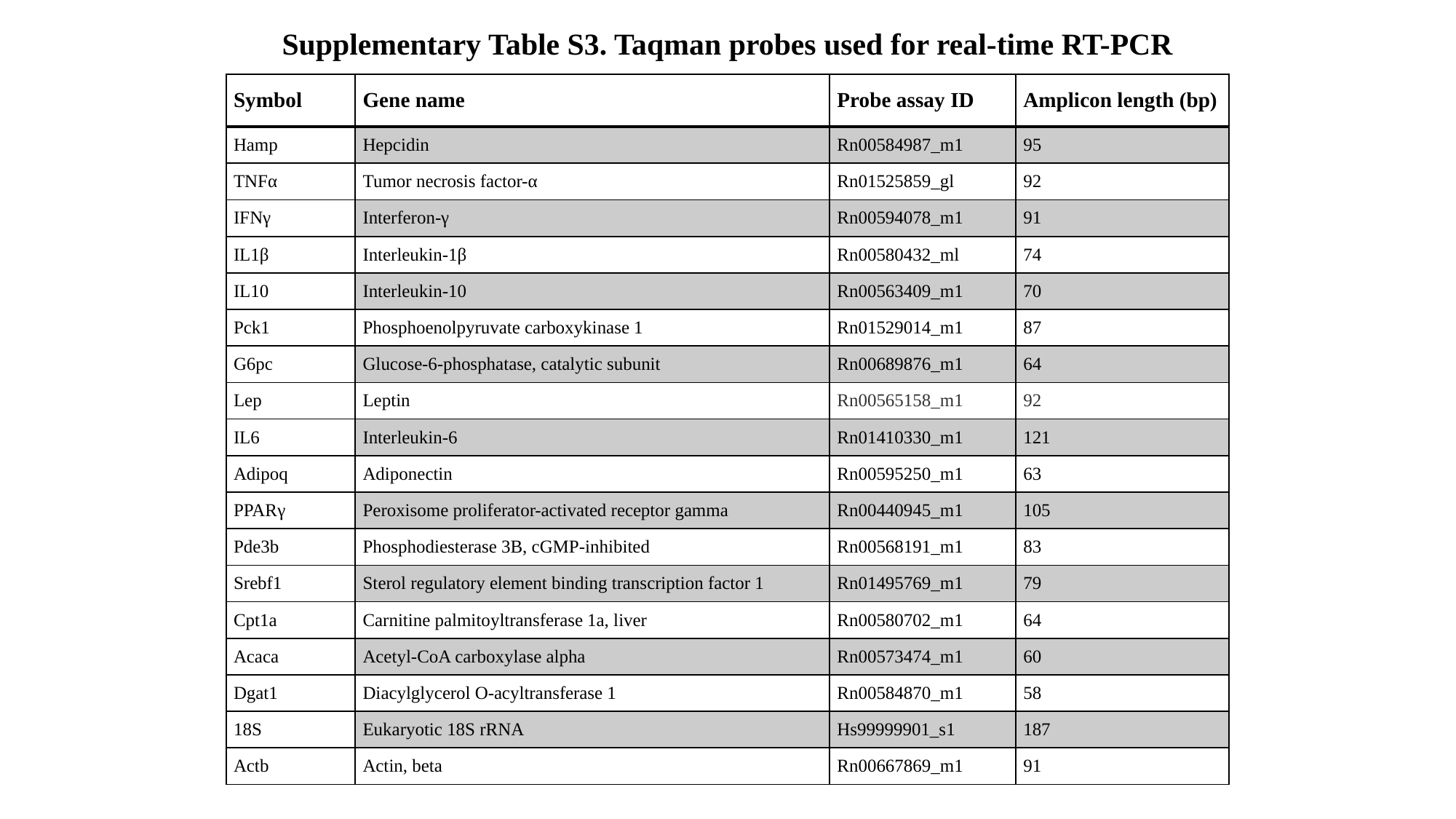

Supplementary Table S3. Taqman probes used for real-time RT-PCR
| Symbol | Gene name | Probe assay ID | Amplicon length (bp) |
| --- | --- | --- | --- |
| Hamp | Hepcidin | Rn00584987\_m1 | 95 |
| TNFα | Tumor necrosis factor-α | Rn01525859\_gl | 92 |
| IFNγ | Interferon-γ | Rn00594078\_m1 | 91 |
| IL1β | Interleukin-1β | Rn00580432\_ml | 74 |
| IL10 | Interleukin-10 | Rn00563409\_m1 | 70 |
| Pck1 | Phosphoenolpyruvate carboxykinase 1 | Rn01529014\_m1 | 87 |
| G6pc | Glucose-6-phosphatase, catalytic subunit | Rn00689876\_m1 | 64 |
| Lep | Leptin | Rn00565158\_m1 | 92 |
| IL6 | Interleukin-6 | Rn01410330\_m1 | 121 |
| Adipoq | Adiponectin | Rn00595250\_m1 | 63 |
| PPARγ | Peroxisome proliferator-activated receptor gamma | Rn00440945\_m1 | 105 |
| Pde3b | Phosphodiesterase 3B, cGMP-inhibited | Rn00568191\_m1 | 83 |
| Srebf1 | Sterol regulatory element binding transcription factor 1 | Rn01495769\_m1 | 79 |
| Cpt1a | Carnitine palmitoyltransferase 1a, liver | Rn00580702\_m1 | 64 |
| Acaca | Acetyl-CoA carboxylase alpha | Rn00573474\_m1 | 60 |
| Dgat1 | Diacylglycerol O-acyltransferase 1 | Rn00584870\_m1 | 58 |
| 18S | Eukaryotic 18S rRNA | Hs99999901\_s1 | 187 |
| Actb | Actin, beta | Rn00667869\_m1 | 91 |

## Slide 13
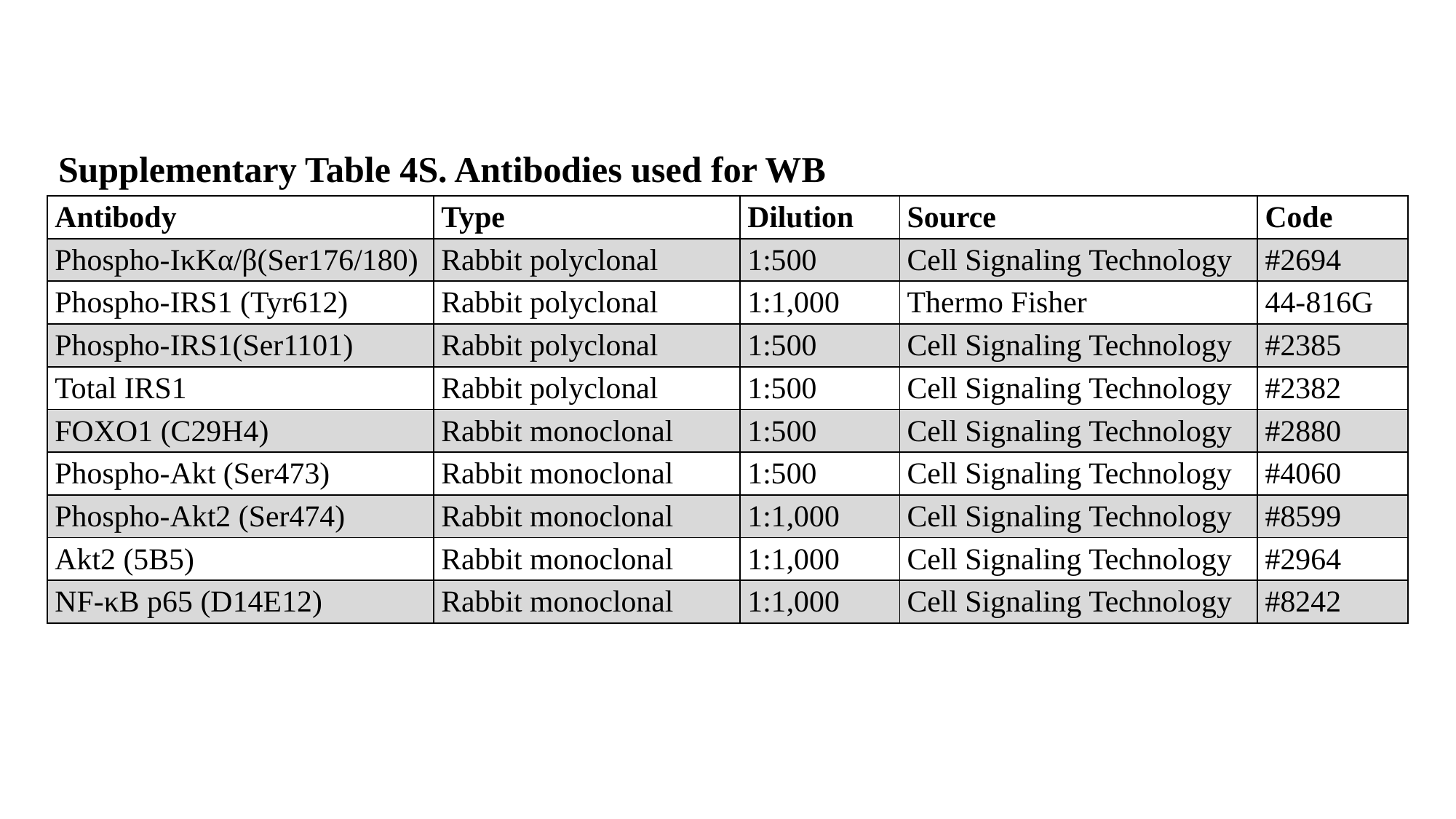

Supplementary Table 4S. Antibodies used for WB
| Antibody | Type | Dilution | Source | Code |
| --- | --- | --- | --- | --- |
| Phospho-IκKα/β(Ser176/180) | Rabbit polyclonal | 1:500 | Cell Signaling Technology | #2694 |
| Phospho-IRS1 (Tyr612) | Rabbit polyclonal | 1:1,000 | Thermo Fisher | 44-816G |
| Phospho-IRS1(Ser1101) | Rabbit polyclonal | 1:500 | Cell Signaling Technology | #2385 |
| Total IRS1 | Rabbit polyclonal | 1:500 | Cell Signaling Technology | #2382 |
| FOXO1 (C29H4) | Rabbit monoclonal | 1:500 | Cell Signaling Technology | #2880 |
| Phospho-Akt (Ser473) | Rabbit monoclonal | 1:500 | Cell Signaling Technology | #4060 |
| Phospho-Akt2 (Ser474) | Rabbit monoclonal | 1:1,000 | Cell Signaling Technology | #8599 |
| Akt2 (5B5) | Rabbit monoclonal | 1:1,000 | Cell Signaling Technology | #2964 |
| NF-κB p65 (D14E12) | Rabbit monoclonal | 1:1,000 | Cell Signaling Technology | #8242 |
